# STING/type I interferon pathway is required for antigen-containing PLGA nanoparticle- and apoptotic cell–induced CD4+ T cell tolerance

**DOI:** 10.1126/sciadv.adv8860

**Published:** 2026-01-02

**Authors:** Joseph R. Podojil, Andrew C. Cogswell, Tobias Neef, Ming-Yi Chiang, Sara A. Beddow, Gabriel Arellano, Sandeep Kakade, Derrick P. McCarthy, Adam Elhofy, Chris T. Harp, Mairah Khan, Joshua J. Meeks, Dan Xu, Lonnie D. Shea, Stephen D. Miller

**Affiliations:** ^1^ Department of Microbiology-Immunology, Northwestern University, Chicago, IL, USA.; ^2^ Center for Human Immunobiology, Northwestern University, Chicago, IL, USA.; ^3^ Cour Pharmaceutical Development Company, Skokie, IL, USA.; ^4^ Urology Feinberg School of Medicine, Northwestern University, Chicago, IL, USA.; ^5^ Department of Biomedical Engineering, University of Michigan, Ann Arbor, MI, USA.

## Abstract

Autoreactive CD4^+^ T cell infiltration, tissue destruction, and spread epitope–specific CD4^+^ T cell activation underly CD4^+^ T cell–mediated autoimmune disease pathogenesis. Here, we identify previously unknown pathways required for antigen (Ag)–specific tolerogenic immune-modifying particle/Cour nanoparticle (TIMP/CNP)–induced tolerance. The data show that myeloid cells phagocytose CNPs, undergo apoptosis, and release oxidized DNA [8-hydroxy-2′-deoxyguanosine (8-OHG)]. Subsequently, Ag-specific CNP treatment increases the number of PD-L1^+^ cDC2 dendritic cells and the number of FoxP3^+^, CTLA-4^+^, PD-1^+^, and IL-10^+^ regulatory CD4^+^ T cells via a stimulator of interferon genes (STING)/interferon-α/β receptor (IFNAR)–dependent pathway. In addition, these same pathways were found to be required for both Ag-coupled apoptotic leukocyte–induced and Ag-coupled red blood cell treatment–induced CD4^+^ T cell tolerance. Together, these results show that Ag-specific tolerance induced by the presence of apoptotic cells, and by CNP-induced apoptosis, requires the STING/IFNAR pathway, thereby illustrating a previously unknown function of this pathway.

## Teaser

STING/IFNAR is required for both antigen-specific biodegradable nanoparticle- and apoptotic cell–induced immune tolerance in vivo.

## INTRODUCTION

Autoimmune diseases are characterized by a failure of immune regulation, resulting in the destruction of self-tissue by autoreactive T cells. Thus, numerous immunosuppressive therapies targeting T cell activation, migration, and function are standard practice for treating autoimmune diseases (*1*). We previously reported that tolerance induced by intravenous administration of proteins/peptides cross-linked, using 1-ethyl-3-(3-dimethylaminopropyl)carbodiimide (EDC), to syngeneic apoptotic splenic leukocytes or red blood cells (RBCs) safely and efficiently induced antigen (Ag)–specific immune tolerance. Treatment has been shown to be functional for both the prevention and therapeutic treatment of T helper type 1 cell (T_H_1 cell)– and T_H_17 cell–mediated autoimmune diseases (*2*–*4*). This strategy proved to be both safe and able to decrease Ag-specific CD4^+^ T cell responses to myelin peptides ex vivo in a phase 1 clinical trial in patients with multiple sclerosis (MS) (*5*). However, the manufacturing process for this tolerance inducing approach is complex and expensive because autologous leukocytes must be isolated and peptide coupled under good manufacturing practice conditions before reinfusion. To overcome the cost and complexity of this cell-based approach, we established a tolerance methodology using intravenous delivery of proteins/peptides associated with 500-nm carboxylated “biodegradable” poly(d,l-lactic-*co*-glycolic acid) (PLGA) nanoparticles, i.e., tolerogenic immune-modifying particles (TIMPs), also referred to as Cour nanoparticles (CNPs). CNPs serve as an effective method for inducing robust Ag-specific tolerance for the prevention and treatment of the experimental autoimmune encephalomyelitis (EAE) model of MS and the nonobese diabetic (NOD) model of type 1 diabetes (T1D) when administered at the onset or peak of acute disease. In addition, administration of Ag-specific CNPs during EAE remission minimizes epitope spreading and subsequent disease relapses (*6*–*8*). Critically, these preclinical findings have been successfully translated to a phase 1/2a clinical trial. Patients with celiac disease treated with TIMP-gliadin (TIMP-GLIA/TAK-101/CNP-101) or placebo treatment were later orally challenged with gluten. TIMP-GLIA treatment was safe and induced a significant decrease in gliadin-specific interferon-γ (IFN-γ) secretion by T cells upon ex vivo antigen challenge and prevented oral gluten challenge–induced pathologic alterations in the small bowel (*9*).

Although our published findings on both Ag-coupled leukocyte and CNP therapies showed a significant decrease in Ag-specific effector CD4^+^ T cells concomitant with an increase in the number of regulatory FoxP3^+^ CD4^+^ T cells (T_reg_ cells) (*10*), the cellular and molecular mechanisms of action underlying these immunological alterations was not understood. We have recently published that the cyclic guanosine monophosphate–adenosine monophosphate synthase (cGAS)/stimulator of interferon genes (STING) pathway was required for the functional activity of unloaded PLGA nanoparticles (ONP-302) for modulating tumor immunity (*11*). cGAS/STING was initially found as an innate immune sensor that responds to cytosolic DNA in the context of bacterial and viral infections; Initially cGAS binds to cytosolic double-stranded DNA (dsDNA) to form 2′,3′-cyclic guanosine monophosphate–adenosine monophosphate (cGAMP), which then signals through STING (*12*, *13*). Recent reports have shown that activation of the STING pathway using multiple doses of microparticles loaded with STING agonists delayed the onset of EAE in an interleukin-10 (IL-10)– and transforming growth factor–β (TGF-β)–dependent manner. However, this delay in onset of EAE was not Ag-specific and daily dosing was required for treatment efficacy (*14*). Furthermore, treatment of NOD mice with STING agonists delays onset and decreases the incidence of T1D (*15*). In addition, STING-deficient NOD mice have an increased number of diabetogenic Ag-specific CD4^+^ T cells within the pancreas and increased disease incidence compared to wild-type (WT) NOD mice (*16*). These findings suggest a putative role for STING in immunoregulation. In the present study, we show the critical requirement of the STING pathway for induction of tolerance following the treatment of mice with Ag-specific CNPs, Ag-coupled apoptotic splenocytes, and Ag-coupled RBCs.

Secretion of type I IFNs is a downstream consequence of STING signaling (*13*, *17*–*19*). Type I IFNs consist of multiple families and subtypes with wide-ranging pleiotropic effects depending on the context in which they are produced (*17*). IFN-β has been used as a frontline treatment for MS since 1993 (*20*, *21*). IFN-β therapy for MS is reported to work through several immunoregulatory mechanisms including increasing anti-inflammatory cytokines (*22*), down-regulation of pro-inflammatory T_H_1/T_H_17 cytokines (*23*, *24*), reducing T cell activation and infiltration to the central nervous system (CNS) (*25*), and inducing neuroantigen-specific T_reg_ cells (*26*). A prior work by our laboratory identified a robust type I IFN genetic signature induced via the STING pathway in mice treated with Unloaded CNPs/ONP-302 (*11*). It has recently been found that type I IFN signaling in thymic antigen-presenting cells (APCs) is vital for establishing central tolerance by T_reg_ cells (*27*). Because the only difference between Unloaded and Ag-encapsulating CNPs is the encapsulated peptide or protein Ag(s), we hypothesized that the STING pathway may be required for Ag-specific tolerance induction.

Here for the first time to our knowledge, we show that Ag-specific tolerance induced by CNP treatment uses identical mechanisms of T_reg_ cell–dependent control of autoreactive T cells as that activated by EDC Ag-coupled apoptotic leukocyte/RBC-induced tolerance. Following the treatment of mice with Ag-specific CNP, the CNPs are phagocytized by myeloid cells, which then undergo apoptosis. CNP-induced T cell tolerance is a highly active and coordinated process inducing increased expression of regulatory receptors/ligands, such as programmed death-1 (PD-1), programmed death-ligand 1 (PD-L1), cytotoxic T-lymphocyte-associated protein 4 (CTLA-4), and IL-10, as well as significant expansion of Ag-specific T_reg_ cells and Tr1 T cells. CNP-induced tolerance depends on T_reg_ cell induction and function as T_reg_ cell inactivation or regulatory protein blockade inhibits Ag-specific CNP tolerance. Last, we show that the coordination of Ag-specific tolerance induced by both CNPs and Ag-coupled apoptotic leukocytes/RBCs is dependent on the generation of oxidized DNA [8-hydroxy-2′-deoxyguanosine (8-OHG)] and subsequent activation of the STING pathway and local secretion of type I IFNs. The present findings thus elucidate the molecular pathways involved in a clinically translatable therapy. In addition, these findings identify a previously unidentified role for the STING pathway following the phagocytosis of apoptotic cells for the induction and maintenance of Ag-specific peripheral tolerance in vivo. CNP-induced tolerance thus recapitulates the natural mechanism by which self-tolerance is induced and maintained in the hematopoietic system, which is subject to massive daily cellular turnover.

## RESULTS

### CNP-PLP_139-151_ treatment expands a regulatory population of antigen-specific T cells

**Fig. 1.**
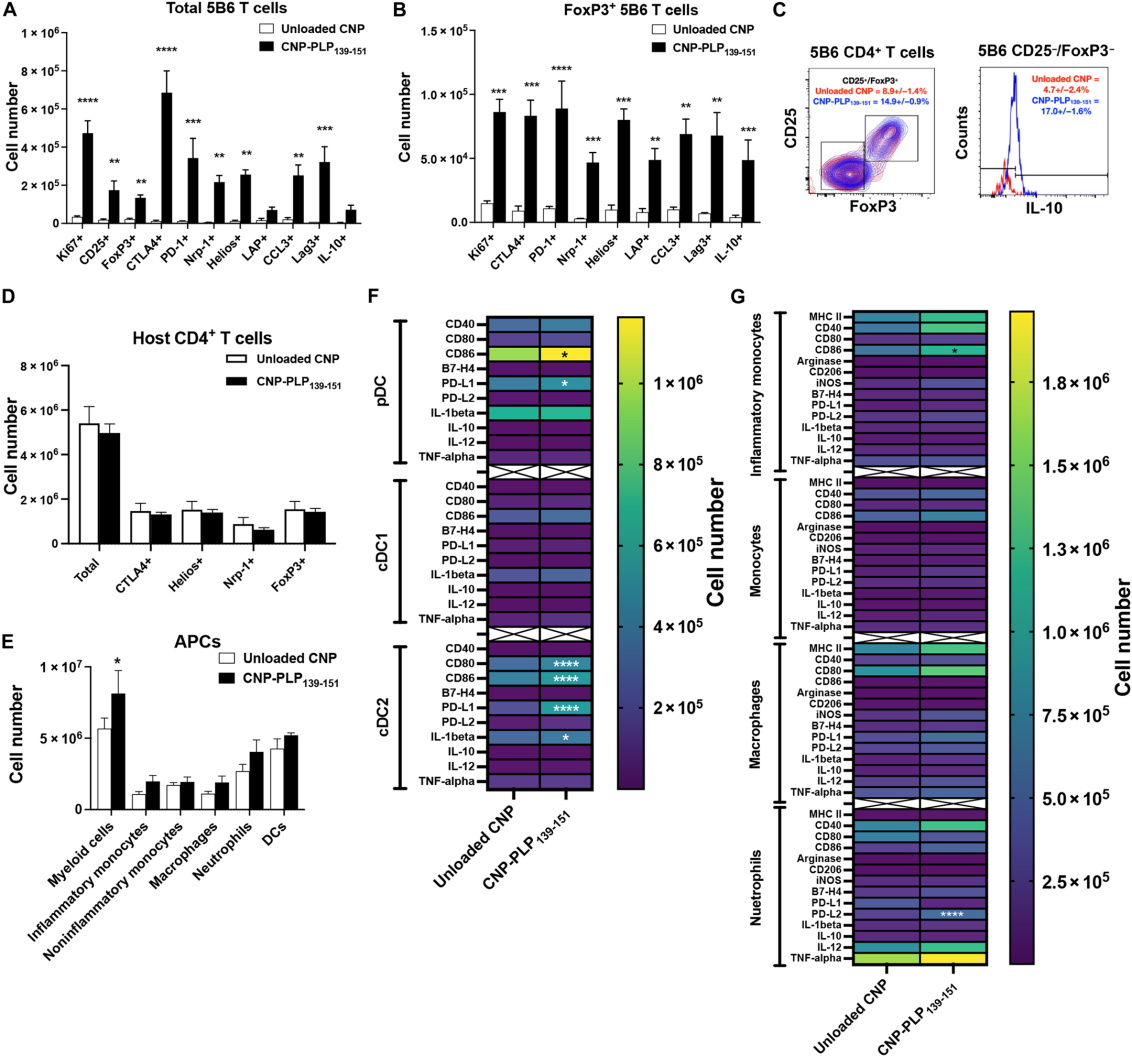
CNP-PLP_139-151_ treatment increases the number of Ag-specific T_reg_ cells within the spleen. SJL/J (CD90.2^+^) mice (7- to 8-week-old female; *n* = 4 per treatment group) received purified 5B6 CD90.1^+^ CD4^+^ T cells via intravenous injection on Day 0, mice were treated as indicated on Day 2, and the spleen was analyzed by flow cytometry on Day 7. The number of total 5B6 CD4^+^ T cells (**A**) and FoxP3^+^ 5B6 CD4^+^ T cells is presented (**B**), as well as the percentage of CD25^+^/FoxP3^+^ 5B6 CD4^+^ T cells (Unloaded CNP in red and CNP-PLP_139-151_ in blue) and the percentage of IL-10^+^ CD25^−^/FoxP3^−^ 5B6 CD4^+^ T cells (**C**). The number of host CD4^+^ T cells expressing regulatory markers is presented (**D**). The number of total myeloid cells inflammatory monocytes, noninflammatory monocytes, macrophages, and neutrophils is presented posttreatment (**E**), as are the number of DCs (**F**) and myeloid cells (**G**) expressing the respective markers. One representative experiment of three is presented. Asterisks indicate a statistically significant difference as compared to the Unloaded CNP treatment group, *P < 0.05, **P < 0.01, ***P < 0.001, and ****P < 0.0001, respectively.

Our previous results showed that Ag-specific CNP treatment increased the number of T_reg_ cells and decreased the number of effector CD4^+^ T cells within the disease-associated target tissue (*8*, *28*). However, the phenotype of the resultant Ag-specific CD4^+^ T cells was not established, and it was not determined whether the number of Ag-specific T_reg_ cells was increased following Ag-specific CNP treatment. Because WT SJL/J (CD90.2/Thy1.2^+^) mice have a limited precursor frequency of EAE disease epitope-specific (PLP_139-151_) CD4^+^ T cells, supplementation with CD90.1^+^ 5B6 transgenic T cells enabled the tracking of the number and phenotype of PLP_139-151_-specific CD4^+^ T cells in response to treatment with Unloaded CNP versus CNP-PLP_139-151_. On Day 5 post–CNP treatment, spleens (Fig. 1) and livers (fig. S1) were collected, and the total number and phenotype of 5B6 CD4^+^ T cells assessed. CNP-PLP_139-151_ treatment expanded the total number of 5B6 CD4^+^ T cells that were proliferative (Ki67^+^) and expressed FoxP3, CTLA-4, and other regulatory/anti-inflammatory markers, as compared to mice treated with Unloaded CNP (Fig. 1, A and B). In addition, the percentage of CD25^+^ FoxP3^+^ 5B6 CD4^+^ T cells was significantly increased in the CNP-PLP_139-151_–treated mice. When the CD25^−^/FoxP3^−^ population of 5B6 CD4^+^ T cells was analyzed further for IL-10 expression, the data show that CNP-PLP_139-151_ treatment also increased the percentage of IL-10^+^ cells, indicating that Tr1 cells are increased (Fig. 1C). Similar changes in the number and phenotype of the transferred 5B6 CD4^+^ T cells were found within the liver (fig. S1, B and C). In contrast, host CD90.2^+^ CD4^+^ T cells did not show a significant change in the total numbers of spleen cells that were CTLA-4^+^, Helios^+^, Nrp-1^+^, or FoxP3^+^, illustrating the Ag specificity of CNP-PLP_139-151_ treatment (Fig. 1D). This is in contrast to Ag-specific CNP treatment during EAE (*7*), celiac disease (*29*), and T1D (*8*), where host Ag-specific CD4^+^ T cells would be expanded due to the ongoing immune response, and consequently, Ag-specific CNP treatment results in a significant increase in the number of host CTLA-4^+^, PD-1^+^, and FoxP3^+^ CD4^+^ T cells present within the spleen.

We also examined the effects of Ag-specific CNP treatment on APC populations, as compared to mice treated with Unloaded CNP. To determine whether the presence of cognate Ag within the CNP results in an alteration in the APC present within the spleen and liver, the phenotype and number of pDCs (CD11c^+^/MHCII^+^/B220^+^), cDC1s (CD11c^+^/MHCII^+^/B220^−^/CD8^+^/XCR1^+/−^), cDC2s (CD11c^+^/MHCII^+^/B220^−^/CD11b^+^/SIRPα^+^), monocytes (CD11c^−^/CD11b^+^/Ly6C^lo^/Ly6G^−^/F4-80^−^), inflammatory monocytes (CD11c^−^/CD11b^+^/Ly6C^+^/Ly6G^−^/F4-80^−^), macrophages (CD11c^−^/CD11b^+^/Ly6C^lo/+^/Ly6G^−^/F4-80^+^), and neutrophils (CD11c^−^/CD11b^+^/Ly6C^+^/Ly6G^+^) were assessed by flow cytometry. CNP-PLP_139-151_ treatment resulted in a significant increase in the number of total CD11b^+^/CD11c^−^ myeloid cells in the spleen (Fig. 1E) and liver (fig. S1D) and increased the total number of dendritic cells (DCs) in the liver (fig. S1D). When these cell populations were further phenotyped for subpopulations, activation markers, cytokines, and regulatory markers, CNP-PLP_139-151_ treatment was found to increase the number of CD86^+^ and PD-L1^+^ pDCs and cDC2s within the spleen while also increasing the number of CD80^+^ cDC2s (Fig. 1F). Similar increases in the number of CD80^+^, CD86^+^, and PD-L1^+^ pDCs, cDC1, and cDC2s was found within the liver (fig. S1E). In addition, the numbers of CD86^+^ inflammatory monocytes and PD-L2^+^ neutrophils were also increased in the spleen (Fig. 1G) and liver (fig. S1F). Thus, Ag-specific CNP-PLP_139-151_ treatment significantly altered the number and phenotype of the DCs present within the spleen and liver. Given that both Unloaded CNPs and CNP-PLP_139-151_ are phagocytosed by splenic and liver myeloid cells (*6*, *30*), the phenotypic differences shown are likely due to APC–T cell cognate interactions.

### CNP-PLP_139-151_ treatment alters the transcriptome within antigen-specific T cells

**Fig. 2.**
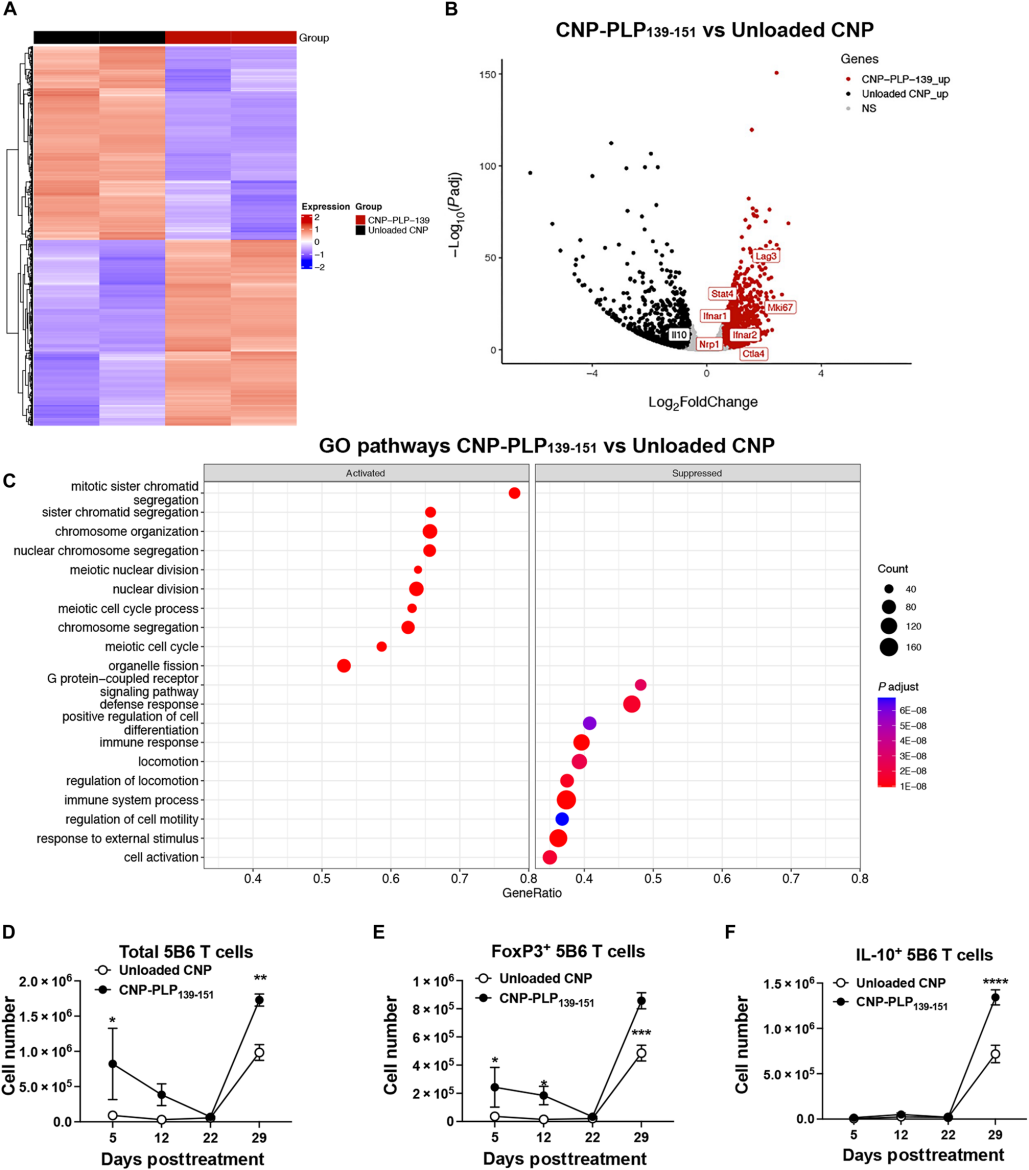
CNP-PLP_139-151_ treatment actively induces a proliferative T_reg_ cell phenotype. SJL/J (CD90.2^+^) mice (7- to 8-week-old female; *n* = 4 per treatment group) received 5B6 CD90.1^+^ CD4^+^ T cells via intravenous injection on Day 0, mice were treated as indicated on Day 2, and 5B6 CD90.1^+^ CD4^+^ T cells were sort purified form total splenocytes and total RNA extracted on Day 5. The data are presented as a heatmap showing the differential expression and clustering (**A**), volcano plot showing the differential expression of genes (**B**), and gene pathway analysis (**C**). Gene ratio is the gene sets of interest/total genes in the set. Total Count is the number of genes belonging to the gene set. Bonferroni correction applied for pathway analyses, *P*adj < 0.05. In separate cohorts of recipient mice, spleens were collected on Days 5, 12, 22, and 29. For the mice analyzed on Day 29, these mice were primed with PLP_139-151_/CFA on Day 22. The number of total 5B6 CD4^+^ T cells (**D**), FoxP3^+^ 5B6 CD4^+^ T cells (**E**), and IL-10^+^ 5B6 CD4^+^ T cells (**F**) within the spleen per mouse was quantified over time. One representative experiment of two is presented. Asterisks indicate a statistically significant difference as compared to the Unloaded CNP treatment group, *P < 0.01, **P < 0.001, ***P < 0.0001, and ****P < 0.0001, respectively.

The findings above suggest that Ag-specific CNP treatment expands the number of Ag-specific CD4^+^ T_reg_ cells and induces the expression of regulatory molecules on splenic and liver DCs. To better understand the molecular events underlying Ag-specific CNP-induced tolerance, we determined the transcriptional profile of 5B6 CD4^+^ T cells postdosing with CNP-PLP_139-151_. 5B6 CD4^+^ T cells were adoptively transferred into naïve SJL/J mice, which were treated with either Unloaded CNP or CNP-PLP_139-151_ on Day 2. On Day 5, spleens were collected, the CD90.1-expressing 5B6 CD4^+^ T cells were sort purified, and total RNA was extracted. Transcript expression of 5B6 CD4^+^ T cells from mice treated with CNP-PLP_139-151_ differed significantly from 5B6 CD4^+^ T cells from mice treated with Unloaded CNP (Fig. 2A), showing increases in *Lag3*, *IFNAR1*, *IFNAR2*, *CTLA-4*, and *Nrp-1* (Fig. 2B). Pathway analysis further confirmed that CNP-PLP_139-151_ treatment significantly enriched mitotic-associated transcripts while decreasing defense response– and cellular locomotion–associated transcripts (Fig. 2C). Together, the findings suggest that a process of cellular activation within the Ag-specific CD4^+^ T cell is induced post–Ag-specific CNP treatment (Fig. 2, B and C), thereby supporting the phenotypic data shown in Fig. 1. To determine whether Ag-specific CNP treatment induced a stable phenotype, the numbers and phenotype of 5B6 CD4^+^ T cells from mice treated with Unloaded CNP or CNP-PLP_139-151_ was determined on Days 5, 12, and 22 post–5B6 CD4^+^ T cell transfer (representing Days 3, 10, and 20 post–Unloaded CNP versus CNP-PLP_139-151_ treatment). As shown in Fig. 2 (D to F), CNP-PLP_139-151_ treatment rapidly increased the number of both total 5B6 CD4^+^ T cells and FoxP3^+^ 5B6 CD4^+^ T cells, which declined by Day 22 (20 days post–CNP-PLP_139-151_). This decline was expected as, in the absence of continued Ag stimulation within the spleen, the Ag-specific CD4^+^ T cells should traffic out of the spleen, thereby decreasing spleen numbers over time. However, to assess whether the 5B6 CD4^+^ T cells were still present within the mice and maintained a regulatory phenotype, a cohort of Unloaded CNP versus CNP-PLP_139-151_–treated mice were primed with PLP_139-151_/complete Freund’s adjuvant (CFA) on Day 22. On Day 29 (7 days after PLP_139-151_/CFA priming), spleens were collected, and the number of total 5B6 CD4^+^ T cells (Fig. 2D), FoxP3^+^ 5B6 CD4^+^ T cells (Fig. 2E), and IL-10^+^ 5B6 CD4^+^ T cells (Fig. 2F) was determined. Significantly higher numbers of total, FoxP3^+^, and IL-10^+^ 5B6 CD4^+^ T cells were found within CNP-PLP_139-151_–treated mice, suggesting that Ag-specific CNP treatment induces a stable Ag-specific regulatory phenotype.

### Functional T_reg_ cells are required for effective tolerance induction

**Fig. 3.**
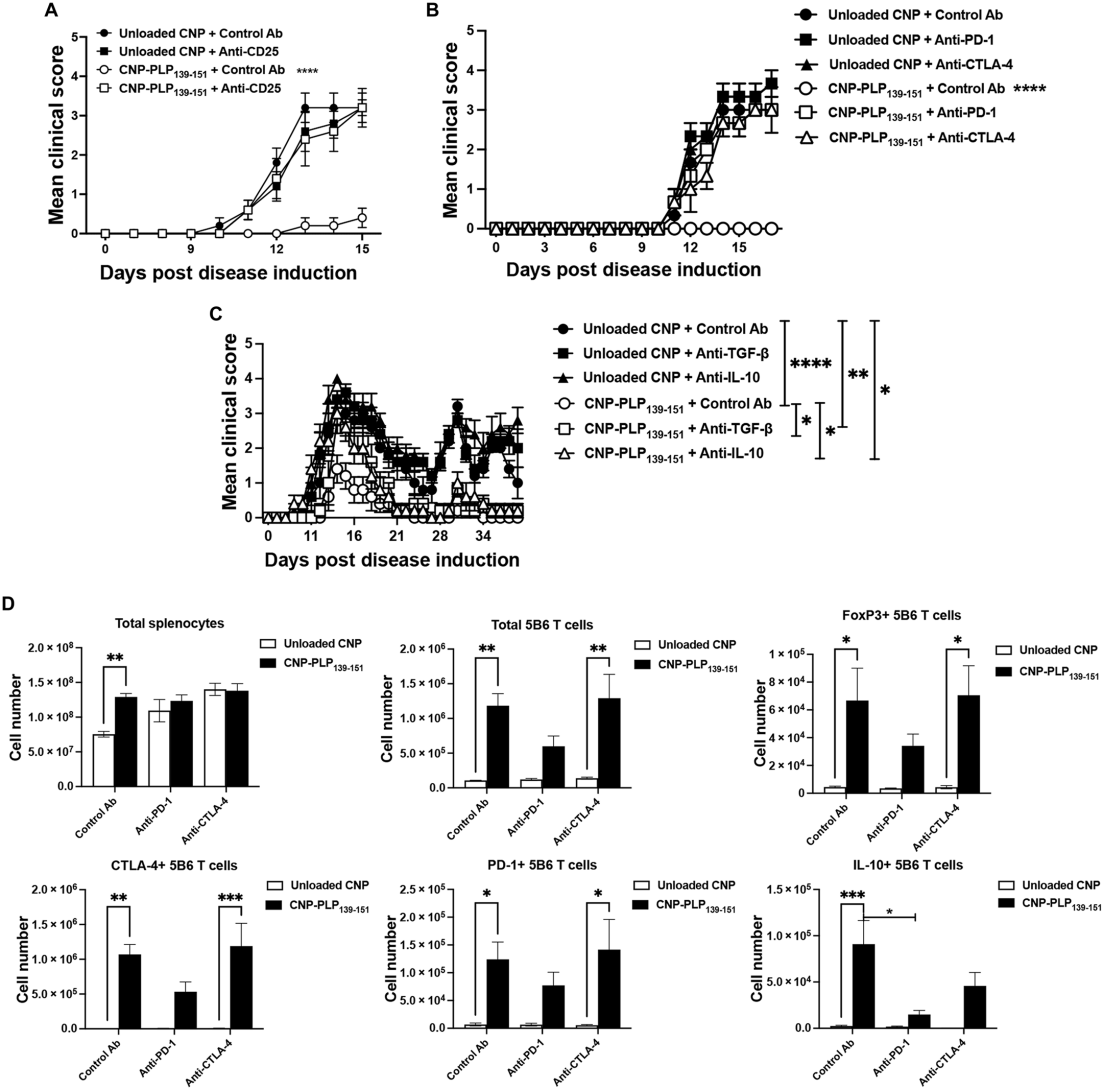
Functional T_reg_ cells are required to induce tolerance with CNP-PLP_139-151_ treatment in an EAE priming model. SJL/J mice (7- to 8-week-old female; *n* = 5 per treatment group) were primed with PLP_139-151_/CFA and treated as indicated on Day 0. On Days 5 and 7, mice were treated with either Control Ab or anti-CD25 mAb (500 μg per dose) (**A**), anti–PD-1 mAb, anti–CTLA-4 mAb (**B**), or anti–TGF-β mAb, anti–IL-10 mAb (**C**) on Days 0, 2, 4, 7, 9, and 11 (100 μg per dose) via intraperitoneal injection. The mice were followed for disease. Alternatively, similar analysis to Fig. 1A was completed in the presence of Control Ab, anti–PD-1 mAb, or anti–CTLA-4 mAb (100 μg per dose) via intraperitoneal injection on Days 0, 2, 4, and 6. On Day 7, the number of total 5B6 CD4^+^ T cells, FoxP3^+^, CTLA-4^+^, PD-1^+^, and IL-10^+^ 5B6 CD4^+^ T cells was analyzed (**D**). One representative experiment of two is presented. Asterisks indicate a statistically significant difference as compared to the Unloaded CNP treatment group, *P < 0.01, **P < 0.001, ***P < 0.0001, and ****P < 0.0001, respectively.

To determine whether functional T_reg_ cells are required for CNP-PLP_139-151_–induced tolerance, SJL/J mice were treated with either Unloaded CNP or CNP-PLP_139-151_ and primed with PLP_139-151_/CFA on Day 0. The mice were subsequently treated with species- and isotype-matched Control antibody (Ab) versus anti-CD25 monoclonal antibody (mAb) on Days 5 and 7 to deplete/inactivate CD25^+^ T_reg_ cells (*31*). As expected, CNP-PLP_139-151_ treatment prevented EAE development (Fig. 3A), but cotreatment with anti-CD25 mAb resulted in total blockade of the CNP-PLP_139-151_ function, demonstrating that CD25^+^ T_reg_ cells are required for CNP-PLP_139-151_–induced tolerance. At Day 16 post–disease induction, spleens and draining lymph nodes were isolated, and ex vivo recall cultures were performed. CNP-PLP_139-151_ treatment significantly decreased the level of granulocyte-macrophage colony-stimulating factor (GM-CSF) and IL-17 secreted while increasing the level of IL-10. These treatment-associated changes in pro- and anti-inflammatory cytokine secretion were reversed by anti-CD25 mAb treatment (fig. S2A). In addition, inguinal lymph node cells cultured in the presence of PLP_139-151_ secreted a significantly lower level of IFN-γ and IL-17 while secreting more IL-10 following CNP-PLP_139-151_ treatment. Like the spleen, these treatment-associated changes in cytokine secretion were reversed by anti-CD25mAb treatment (fig. S2B).

To determine whether the CNP-PLP_139-151_–induced increase in CTLA-4, PD-1, Lap/TGF-β, and IL-10 (Fig. 1, A and B) is functionally required for the induction of Ag-specific tolerance, the effects of Control Ab, anti–PD-1 mAb, anti–CTLA-4 mAb (Fig. 3B), anti–TGF-β mAb, and anti–IL-10 mAb (Fig. 3C) administered post–PLP_139-151_/CFA-induced EAE induction were determined. Ab blocking of each of these CNP-PLP_139-151_–induced regulatory proteins inhibited CNP-PLP_139-151_–induced tolerance as determined by the disease scores, as well as a loss of CNP-PLP_139-151_–induced decrease in pro-inflammatory cytokines in ex vivo recall responses (fig. S2C). The ability of PD-L1-Ig fusion protein to increase the number of T_reg_ cells has been reported (*32*), so we next determined whether blockade of PD-1 or CTLA-4 would inhibit the CNP-PLP_139-151_–induced increase in the number and phenotype of 5B6 CD4^+^ T cells. Following 5B6 CD4^+^ T cell transfer into naïve SJL/J mice on Day 0, mice were treated with either Unloaded CNP or CNP-PLP_139-151_ on Day 2. Mice were subsequently treated with Control Ab, anti–PD-1 mAb, or anti–CTLA-4 mAb on Days 0, 2, 4, and 6. On Day 7, spleens were collected, and the number and phenotype of the 5B6 CD4^+^ T cells were assessed by flow cytometry. Anti–CTLA-4 mAb (Fig. 3D) did not modulate the number and phenotype of 5B6 CD4^+^ T cells in the spleen. However, anti–PD-1 mAb treatment partially inhibited the CNP-PLP_139-151_–induced increase in the number of 5B6 CD4^+^ T cells present within the spleen, and PD-1 blockade partly blocked the CNP-PLP_139-151_–induced increase in FoxP3, CTLA-4, PD-1, and IL-10 expression (Fig. 3D). Together, these findings suggest that, although CNP-PLP_139-151_–induced tolerance required PD-1, CTLA-4, TGF-β, and IL-10 to significantly decrease disease severity during EAE, there is a differential function of these regulatory molecules with PD-1 involved in the induction and/or expansion of the Ag-specific T_reg_ cells. This latter finding correlates with the Ag-specific CNP-induced increase in PD-L1^+^ DCs posttreatment. In addition, although CTLA-4, TGF-β, and IL-10 appear to not be directly involved in the Ag-specific CNP-induced increase in T_reg_ cells, these CD4^+^ T cell–expressed regulatory proteins are required for treatment-induced T_reg_ cell effector regulatory function.

The ability to induce tolerance via T_reg_ cells specific for one Ag that can prevent disease progression due to other disease-specific Ags has been termed infectious tolerance. We thus asked whether CNP-PLP_139-151_–induced 5B6 CD4^+^ T_reg_ cells were able to inhibit the induction of PLP_178-191_/CFA-induced EAE. 5B6 CD4^+^ T cells were transferred into naïve SJL/J mice that were treated with one, two, or three doses of Unloaded CNP or CNP-PLP_139-151_ on Days 2, 9, and/or 16 post–5B6 T cell transfer. Mice were euthanized on Day 22 posttransfer, and spleens were examined by flow cytometric analysis for numbers of 5B6 CD4^+^ T cells and expression of T_reg_/Tr1 cell markers. Three weekly treatments with CNP-PLP_139-151_ resulted in a statistically significant increase in the number of total CD4^+^ total 5B6 T cells in the spleen and a concordant increase in the number of 5B6 CD4^+^ T cells expressing markers associated with the T_reg_/Tr1 phenotype (FoxP3, IL-10, and PD-1), as compared to mice receiving one or two doses of CNP-PLP_139-151_ (fig. S3A). Correlating with the ability of CNP-PLP_139-151_ redosing to further expand Ag-specific 5B6 CD4^+^ T_reg_ cells, SJL/J mice that received 5B6 CD4^+^ T cells before three weekly doses of CNP-PLP_139-151_ showed a significant decrease in the level of both PLP_139-151_/CFA- and PLP_178-191_/CFA-induced EAE (fig. S3B). In addition, CNP-PLP_139-151_ treatment of mice primed with either PLP_139-151_ or PLP_178-191_ inhibited the secretion of IFN-γ (fig. S3C) and increased the level of IL-10 secreted (fig. S3D). In contrast, CNP-PLP_139-151_ treatment of SJL/J mice, which had not received exogenous 5B6 T cells (to increase the frequency of induced PLP_139-151_-specific T_reg_ cells), only resulted in decreased disease severity in mice primed with PLP_139-151_/CFA but not with PLP_178-191_/CFA. However, CNP-PLP_139-151_ treatment did significantly decrease disease severity during subsequent disease relapses in PLP_178-191_/CFA-primed mice, indicating that CNP-PLP_139-151_ treatment inhibited the subsequent PLP_139-151_-specific spread epitope response and further CNS damage (fig. S3E). Recall cultures on Day 35 post–disease induction showed significantly decreased IFN-γ and increased IL-10 production upon ex vivo stimulation with either PLP_178-191_ and PLP_139-151_ in both normal mice and 5B6 recipients (fig. S3, F and G). In summary, the precursor frequency of PLP_139-151_-specific T cells in CNP-PLP_139-151_–treated WT SJL/J mice was sufficient to induce regulatory cytokine production in this model, but this phenotypic change was not sufficient to modulate acute EAE disease symptoms induced by the noncognate PLP_178-191_ epitope.

### 5B6 CD4^+^ T cell number and phenotype are differentially altered following CNP-PLP_178-191_ versus CNP-PLP_139-151_ treatment in PLP_178-191_/CFA-induced EAE

**Fig. 4.**
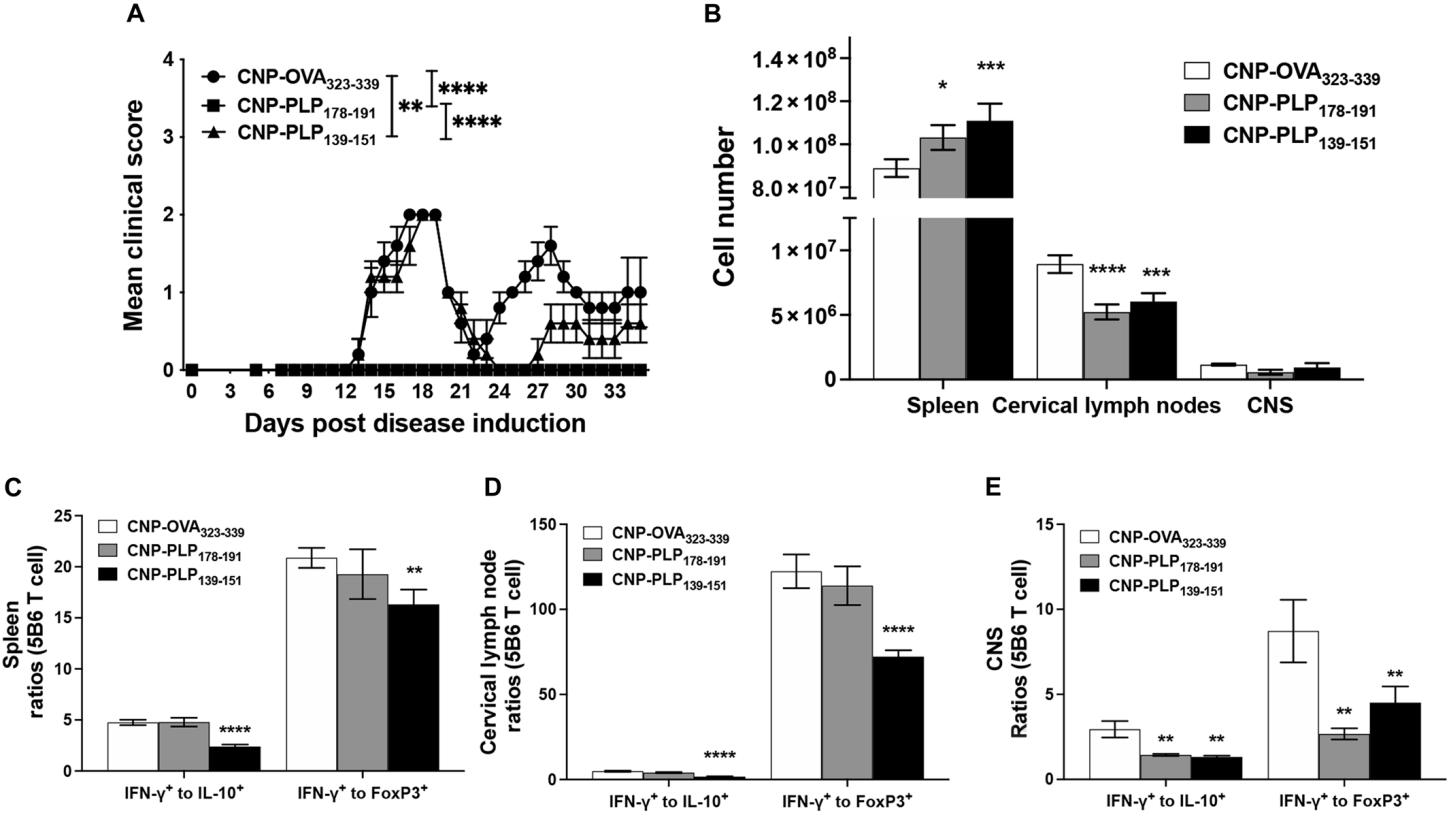
CNP-PLP_178-191_ tolerance induction alters the phenotype of spread epitope–specific T cells. SJL/J (CD90.2^+^) mice (7- to 8-week-old female; *n* = 5 per treatment group) were primed with PLP_178-191_/CFA on Day 0, and mice were followed for disease. On Day 10 post–PLP_178-191_/CFA priming, mice received 3 × 10^6^ sort 5B6 CD90.1^+^ CD4^+^ T cells via intravenous injection. On Day 12, mice were treated with either CNP-OVA_323-339_, CNP-PLP_178-191_, or CNP-PLP_139-151_ (2.5 mg per dose) via intravenous injection. The data are presented as the mean clinical score over time (**A**). On Day 19, spleens, cervical lymph nodes, and CNS were collected from a separate cohort of mice from each treatment group. The total number of CD45^hi^ cells was enumerated (**B**), as were the number of IFN-γ^+^, FoxP3^+^, and IL-10^+^ 5B6 CD4^+^ T cells (fig. S4). The ratio IFN-γ^+^ to IL-10^+^ and IFN-γ^+^ to FoxP3^+^ 5B6 CD4^+^ T cells was calculated per mouse, and the ratios within the spleen (**C**), cervical lymph nodes (**D**), and CNS (**E**) were presented. One representative experiment of two is presented. Asterisks indicate a statistically significant difference as compared to the Unloaded CNP treatment group, *P < 0.05, **P < 0.01, ***P < 0.001, and ****P < 0.0001, respectively.

During relapsing-remitting EAE in SJL/J mice, the acute phase of disease is driven by CD4^+^ T cells specific for the disease-inducing peptide, whereas the primary relapsing disease episode is driven by CD4^+^ T cells specific for the dominant spread epitope, which can be within the same (intramolecular spreading) or a different (intermolecular spreading) myelin protein (*33*, *34*). We previously showed that the spread epitope–specific CD4^+^ T cells are first activated within the CNS by CNS-infiltrating DCs before these spread epitope–specific CD4^+^ T cells are detected within the spleen or cervical lymph nodes (*35*). We thus assessed the effects of a single dose of CNP-PLP_178-191_ versus CNP-PLP_139-151_ on the number and phenotype of the spread epitope–specific CD4^+^ T cells, i.e., transferred PLP_139-151_-specific 5B6 CD4^+^ T cells, in PLP_178-191_/CFA-primed mice. SJL/J mice were thus primed with PLP_178-191_/CFA on Day 0 and received naïve 5B6 CD4^+^ T cells on Day 10. Mice were then randomized into CNP-OVA_323-339_, CNP-PLP_139-151_, or CNP-PLP_178-191_ treatment groups on Day 12. As anticipated, CNP-PLP_178-191_ treatment on Day 12 completely inhibited PLP_178-191_/CFA-induced EAE disease, whereas CNP-PLP_139-151_ treatment significantly inhibited the level of disease severity only during the primary disease relapse, which corresponds to the dominant CD4^+^ T cell responses during the respective phases of disease (Fig. 4A). We next determined the number and phenotype of the spread epitope–specific 5B6 CD4^+^ T cells on Day 17 in the spleens, cervical lymph nodes, and CNS. Both CNP-PLP_139-151_ and CNP-PLP_178-191_ treatment increased cellularity within the spleen and reduced cellularity in the cervical lymph nodes as compared to CNP-OVA_323-339_–treated mice (Fig. 4B). CNP-PLP_139-151_ treatment expanded the total 5B6 CD4^+^ T cells in the spleen as well as IL-10^+^ and Ki67^+^ 5B6 CD4^+^ T cells in the spleen and cervical lymph nodes relative to CNP-OVA_323-339_–treated mice (fig. S4, A and B). CNP-PLP_139-151_ treatment also increased the number of IFN-γ^+^ 5B6 CD4^+^ T cells in the spleen relative to CNP-OVA_323-339_ treatment, showing the ability of Ag-specific treatment to sequester effector CD4^+^ T cells within the spleen (fig. S4A) (*28*). In addition, the number of 5B6 CD4^+^ T cells present within the CNS was not significantly changed between the CNP-OVA_323-339_ and the CNP-PLP_139-151_–treated mice. In contrast, the 5B6 CD4^+^ T cells were not expanded in mice treated with CNP-PLP_178-191_ (fig. S4, A to C). Treatment with CNP-PLP_139-151_, the cognate peptide for the spread epitope–specific 5B6 CD4^+^ T cells, significantly decreased the IFN-γ^+^:IL-10^+^ and IFN-γ^+^:FoxP3^+^ ratios within all tissues (Fig. 4, C to E), whereas CNP-PLP_178-191_ treatment, which prevented acute CNS damage, significantly inhibited the initial activation of 5B6 CD4^+^ T cells, and CNP-PLP_178-191_ treatment was able to induce a decrease in the IFN-γ^+^:IL-10^+^ and IFN-γ^+^:FoxP3^+^ ratios within the CNS. Given the importance of the change in the CD4^+^ T cell phenotype from inflammatory to anti-inflammatory, we determined whether the administration of a Toll-like receptor (TLR) stimulus, i.e., lipopolysaccharide (LPS), would inhibit Ag-specific CNP-induced tolerance. SJL/J mice were treated with either CNP-OVA_323-339_ or CNP-PLP_139-151_ in the absence or presence of LPS (20 μg) on Day −7. The mice were primed with PLP_139-151_/CFA on Day 0 and followed for disease. The data show that concurrent administration of LPS did not inhibit CNP-PLP_139-151_–induced tolerance (fig. S4D). Collectively, the results indicate that, although the spread epitope–specific 5B6 CD4^+^ T cells were able to enter the CNS in CNP-PLP_139-151_–treated mice, these cells displayed a regulatory phenotype. In contrast, CNP-PLP_178-191_ treatment significantly inhibited the acute phase of PLP_178-191_-induced EAE blocking CNS inflammation, thus preventing activation of the spread epitope–specific 5B6 CD4^+^ T cells.

### 8-OHG and apoptosis are consequences of CNP particle uptake and require STING for functional downstream cytokine production

**Fig. 5.**
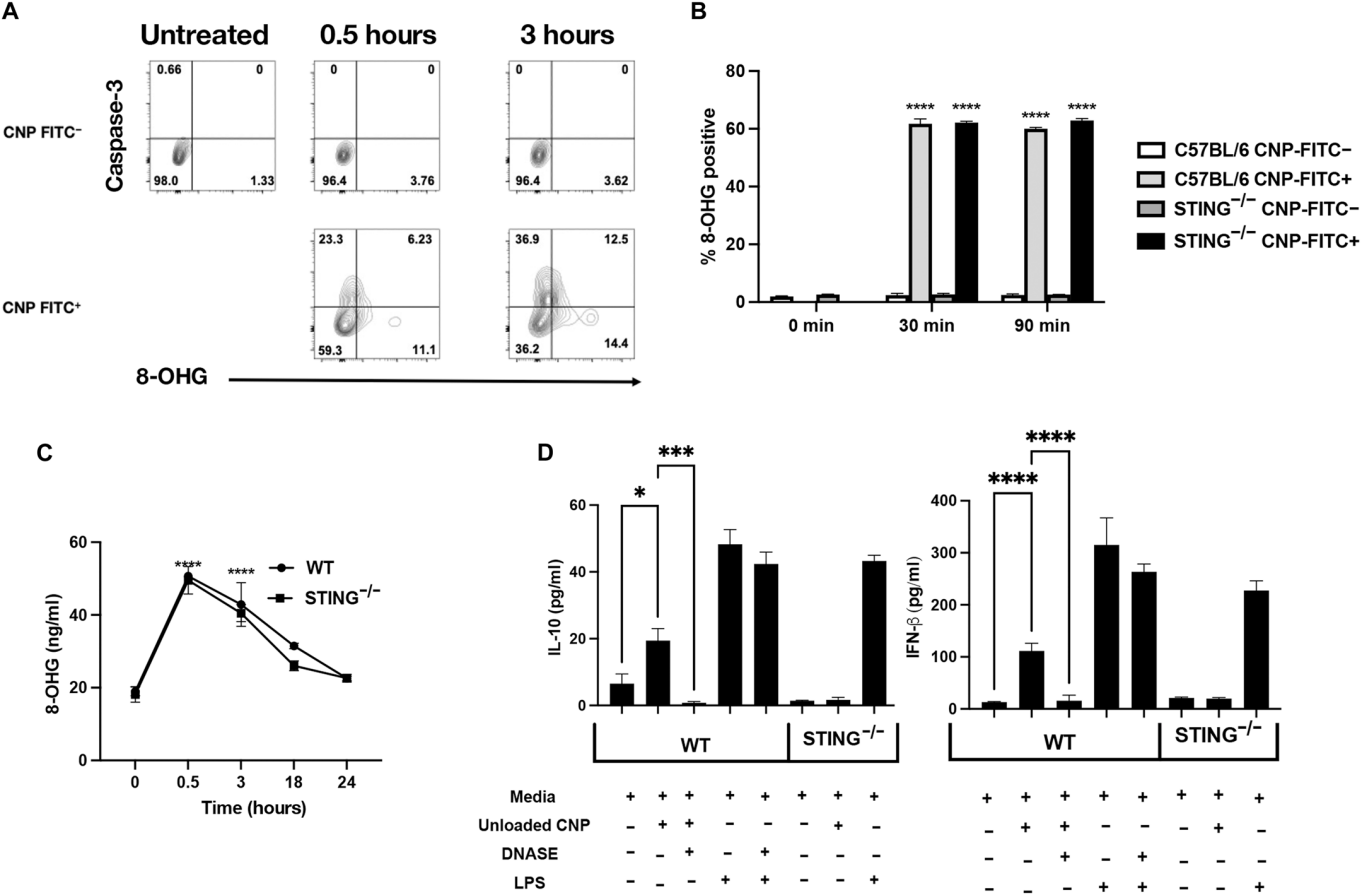
CNP uptake induces 8-OHG in cells, which is necessary for STING signaling. BMDMs were cultured in the presence of CNP-FITC for 0 to 3 hours, and the frequency of CNP-FITC^+^, 8-OHG^+^, and cleaved caspase-3^+^ cells was analyzed (**A**). BMDMs were cultured in the presence of CNP-FITC (5 μg/ml), and the percentage of 8-OHG^+^ cells was analyzed (**B**), as was the level of released 8-OHG (**C**). BMDMs generated from either C57BL/6 or STING^−/−^ mice were cultured in the presence of Unloaded CNP (5 μg/ml) or LPS (1 μg/ml) +/− DNase I (2 U/ml), and the level of secreted IL-10 and IFN-β was assessed at 24 hours postculture (**D**). One representative experiment of two is presented. Asterisks indicate a statistically significant difference as compared to control groups, *P < 0.05, ***P < 0.001, and ****P < 0.0001, respectively.

We recently published that the expression of the cGAS/STING pathway is required for the functional effects of Unloaded CNP in vivo (*11*). Because Ag-encapsulating CNPs are identical with regard to their initial phagocytosis by MARCO^+^ marginal zone macrophages (*6*), with the difference being the encapsulated peptide or protein Ag(s), we hypothesized that the STING pathway may be required for Ag-specific tolerance induction post–Ag-specific CNP treatment. A well-documented ligand of the cGAS/STING pathway is oxidized DNA or 8-OHG (*36*, *37*). Therefore, we wished to determine whether 8-OHG may be the underlying signaling mechanism by which CNPs activate the STING pathway. Initially, we cultured bone marrow–derived myeloid cells (BMDMs) from C57BL/6 mice for 30 and 90 min with fluorescein isothiocyanate (FITC)–labeled CNPs. Bone marrow–derived macrophages were analyzed for the presence or absence of FITC (representing CNP phagocytosis) and for the presence of 8-OHG and cleaved caspase-3 (a surrogate for apoptosis) (Fig. 5A). The data show that BMDMs from both WT C57BL/6 and STING^−/−^ mice phagocytized CNP-FITC, and only the FITC^+^ cells were 8-OHG^+^ at 30 and 90 min (Fig. 5B). Comparable levels of 8-OHG were found in BMDM culture supernatants via enzyme-linked immunosorbent assay (ELISA) from both WT C57BL/6 and STING^−/−^ BMDM for up to 18 hours after CNP addition (Fig. 5C). Therefore, particle phagocytosis leads to the up-regulation of 8-OHG and cleavage of caspase-3, thereby demonstrating that STING is not required for CNP-induced 8-OHG positivity as STING signaling would be downstream of 8-OHG production. In addition, the presence of 8-OHG was independent of Ag loading of the CNP (fig. S5A), and the culture of BMDMs with CNPs induced a concentration-dependent increase in IFN-β secretion. To further test whether the release of 8-OHG was necessary for CNP function, WT C57BL/6 and STING^−/−^ mouse BMDMs were cultured for 18 hours with CNPs in the absence or presence of DNase I, and the level of IL-10 and IFN-β was measured in culture supernatants. As a positive control, LPS treatment of both WT C57BL/6 and STING^−/−^ BMDMs induced secretion of IL-10 and IFN-β in the absence and presence of DNase I. In contrast, CNP-induced secretion of IL-10 and IFN-β by BMDMs was inhibited by the addition of DNase I and the absence of STING expression (Fig. 5D), indicating that uptake of CNPs by BMDMs results in the release of 8-OHG, which activates STING and results in the secretion of IL-10 and IFN-β.

### Antigen presentation after CNP phagocytosis is independent of tolerogenic molecule up-regulation via STING/type I IFNs

**Fig. 6.**
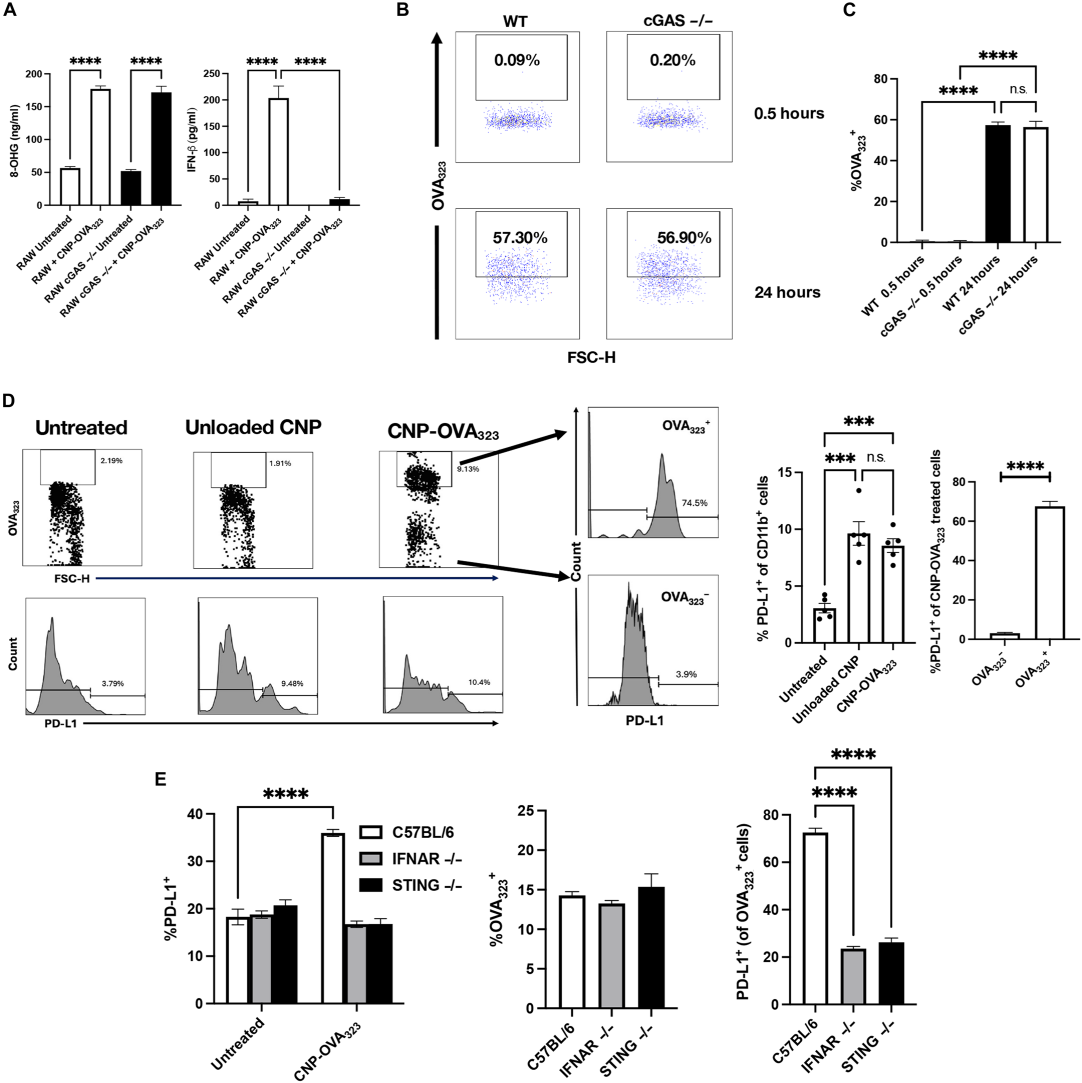
Antigen presentation after CNP phagocytosis is independent of tolerogenic molecule up-regulation via STING/type I IFNs. RAW and RAW cGAS^−/−^ cells were cultured with CNP-OVA_323-339_ (5 μg/ml), and 8-OHG and IFN-β were measured via ELISA (**A**). Representative flow staining of RAW (WT) and RAW cGAS^−/−^ cells for OVA_323-339_ expression 0.5 and 24 hours after treatment with CNP-OVA_323-339_ (**B**). Expression of OVA_323-339_ by WT and cGAS^−/−^ cells after 0.5 and 24 hours of culture with CNP-OVA_323-339_ (**C**). C57BL/6 splenocytes (*n* = 5) were cultured with Unloaded CNP or CNP-OVA_323-339_ (5 μg/ml) for 24 hours, and cells were analyzed for CD11b, PD-L1, and OVA_323-339_ expression (**D**). Expression of PD-L1 and OVA_323-339_ by cDC2s from C57BL/6, IFNAR^−/−^, and STING^−/−^ splenocytes after 24 hours of culture with CNP-OVA_323-339_ (**E**). One representative experiment of two is presented. Asterisks indicate a statistically significant difference as compared to control groups, ***P < 0.001 and ****P < 0.0001, respectively. n.s., not significant.

To further confirm the tolerogenic role of cGAS/STING signaling, we used RAW cells lacking cGAS. Similar to BMDMs from STING^−/−^ mice treated with CNP, RAW cells lacking cGAS up-regulated 8-OHG but did not produce IFN-β after culture in the presence of CNPs (Fig. 6A). Recent reports have indicated that cGAS/STING signaling can enhance the capability of cells to present Ag, and furthermore, type I IFNs are known to significantly up-regulate major histocompatibility complex (MHC)/Ag complexes. Thus, we next asked whether the absence of cGAS affected the ability of RAW cells to present antigen. RAW and RAW cGAS^−/−^ cells were cultured with CNP-OVA_323-339_ and analyzed for surface OVA_323-339_ expression by flow at 0.5 and 24 hours postculture. Both WT and cGAS^−/−^ cells displayed similar levels of antigen presentation (Fig. 6, B and C), demonstrating that the cGAS/STING pathway is not directly involved in presentation of Ag. We hypothesized that the primary function of STING activation following Ag-specific CNP treatment was the modulation of tolerogenic molecule expression (Figs. 1 and 3B). We thus cultured splenocytes from C57BL/6 mice with either Unloaded CNP or CNP-OVA_323-339_ for 24 hours. Although treatment of splenocytes with either Unloaded CNP or CNP-OVA_323-339_ resulted in the up-regulation of PD-L1 on CD11b^+^ cells, only cells from the CNP-OVA_323-339_ treated cultures were double positive for OVA_323-339_ and PD-L1 (Fig. 6D). This suggested that Ag-specific CNP treatment functions by the induction of tolerogenic molecules expressed by Ag-presenting CD11b^+^ cells, i.e., a cell population containing both myeloid cells and cDC2s. To connect the CNP-induced increase in 8-OHG and the requirement for STING signaling to the CNP-induced increase in secreted IFN-β, and Ag presentation by DCs, the same culture system was repeated using WT, STING^−/−^, and IFNAR^−/−^ splenocytes. The gating scheme for assessing OVA_323-339_ presentation and PD-L1 expression by pDCs, cDC1s, and cDC2 is presented in fig. S5D. Although the presence or absence of STING and interferon-α/β receptor (IFNAR) did not alter the percentage of pDCs (fig. S5E), cDC1s (fig. S5E), or cDC2s (Fig. 6E) that were OVA_323-339_^+^, both STING and IFNAR were required for PD-L1 expression and OVA_323-339_ presentation by cDC2s (Fig. 6E). These in vitro data are supportive of the in vivo data presented in Fig. 1F, in that Ag-specific CNP treatment increased PD-L1^+^ cDCs in vivo post–Ag-specific CNP treatment. Together, these data show that CNP treatment induces Ag presentation independent of the STING/IFNAR pathway; however, STING and IFNAR are required for the expression of tolerogenic molecules by cDC2s.

### 8-OHG and STING expression are required for Ag-specific CNP-induced tolerance in vivo

**Fig. 7.**
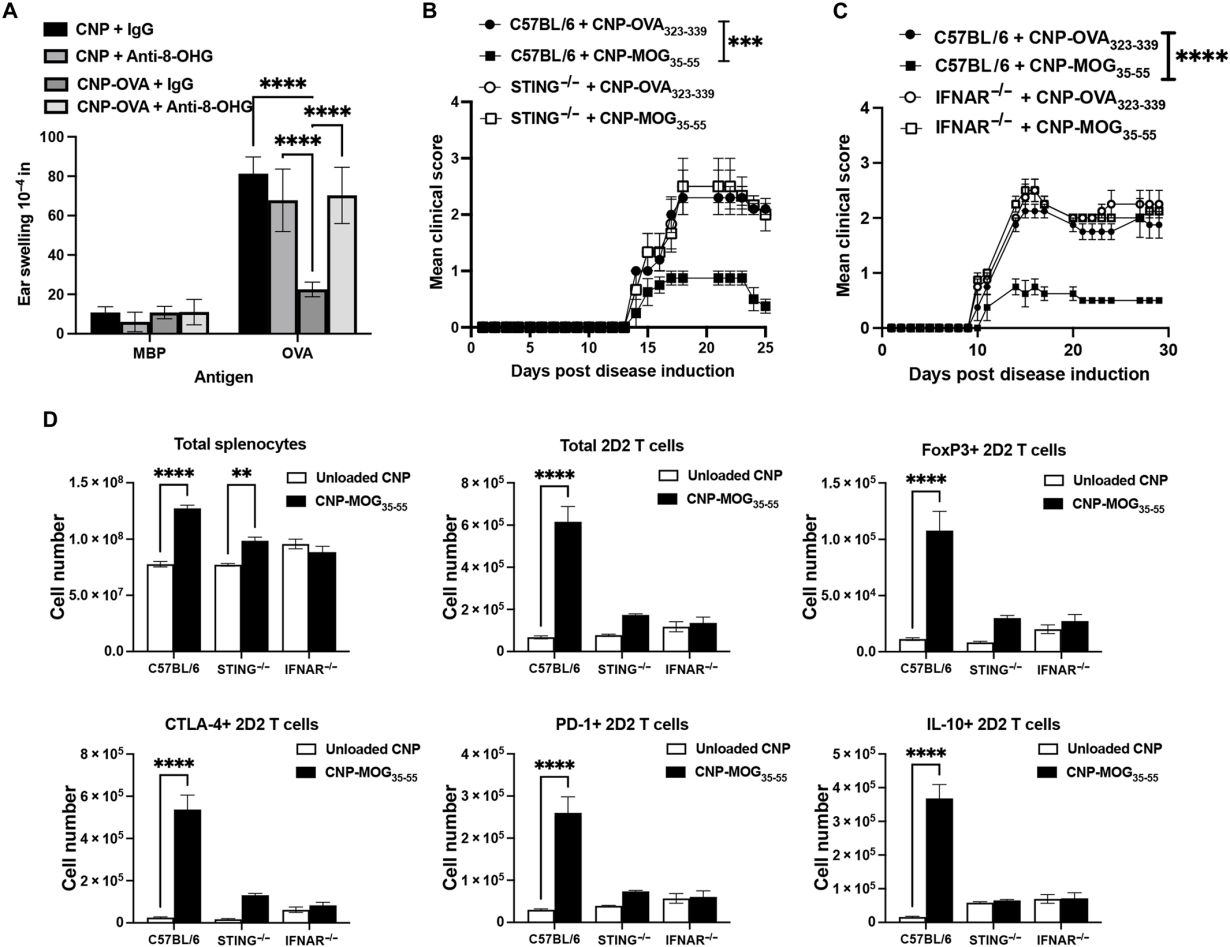
Type I IFN and STING signaling are T cell extrinsic and necessary for CNP-Ag–specific tolerance. C57BL/6 mice (7- to 8-week-old female; *n* = 4 per treatment group) were treated with either a Control Ab or anti–8-OHG (250 μg per dose) intraperitoneally, primed with OVA/CFA, and treated as indicated on Day 0. DTH was completed on Day 14 postpriming (**A**). C57BL/6 and STING^−/−^ mice (7- to 8-week-old female; *n* = 5 per treatment group) were primed with MOG_35-55_/CFA, and mice were treated as indicated on Day 0 and followed for disease (**B**). C57BL/6 and IFNAR^−/−^ mice (7- to 8-week-old female; *n* = 5 per treatment group) were primed with MOG_35-55_/CFA, and mice were treated as indicated on Day 0 and followed for disease (**C**). C57BL/6, STING^−/−^, and IFNAR^−/−^ (CD90.2^+^) mice (7- to 8-week-old female; *n* = 4 per treatment group) received 3 × 10^6^ sort-purified 2D2 CD90.1^+^ CD4^+^ T cells via intravenous injection on Day 0. On Day 2, mice were treated with either Unloaded CNP or CNP-MOG_35-55_ (2.5 mg per dose) via intravenous injection, spleens were collected on Day 7, and splenocytes were analyzed by flow cytometry for the phenotype of the 2D2 CD4^+^ T cells for FoxP3, CTLA-4, PD-1, and IL-10 expression (**D**). One representative experiment of two is presented. Asterisks indicate a statistically significant difference as compared to the Control treatment group, *P < 0.05, **P < 0.01, ***P < 0.001, and ****P < 0.0001, respectively.

Given that 8-OHG activation of STING was required for CNP function in vitro, we next determined whether blockade of 8-OHG in vivo would abrogate Ag-specific CNP-induced tolerance. C57BL/6 mice were treated with Unloaded CNP or CNP-OVA plus a species- and isotype-matched Control Ab or anti–8-OHG on Day 0. The mice were then primed with OVA/CFA on Day 5, and the OVA-specific delayed type hypersensitivity (DTH) response was assessed on Day 19 (14 days after OVA/CFA priming). Blockade of 8-OHG inhibited the ability of CNP-OVA to induce Ag-specific tolerance (Fig. 7A). In addition, ex vivo recall cultures of splenocytes and inguinal lymph node cells showed that, whereas CNP-OVA plus Control Ab treatment significantly reduced the level of IFN-γ, IL-17A, and GM-CSF secreted in response to OVA, in vivo blockade of 8-OHG at the time of CNP-OVA treatment blocked the ability of CNP-OVA to induce Ag-specific tolerance (fig. S5F). Recent studies have shown that STING activation is capable of temporarily ameliorating disease in MOG_35-55_/CFA-induced EAE (*14*, *38*). We thus treated both C57BL/6 WT and STING^−/−^ mice with CNP-OVA_323-339_ versus CNP-MOG_35-55_ and primed the mice with MOG_35-55_/CFA to induce EAE. STING expression was required for CNP-MOG_35-55_–induced tolerance as disease protection was lost in its absence (Fig. 7B). To confirm that this was not due to intrinsic deficiencies between mouse strains, we found no difference in the frequency of T_reg_ cells in the spleen and thymus of 8-week-old STING^−/−^ mice versus WT C57BL/6 controls (fig. S5G). Also, we found no difference between the mouse strains in the frequency of T_reg_ cells induced following the stimulation of splenocytes in the presence of induced T_reg_ cell polarizing conditions in vitro for 3 days (fig. S5G). To further illustrate the requirement for STING activation in the treatment-induced increase in T_reg_ cells, 5B6 splenocytes were cultured for 72 hours in the presence of PLP_139-151_ plus increasing concentrations of the STING agonist G3-YSD. The STING agonist increased the frequency of T_reg_ cells in a concentration-dependent manner (fig. S5H). These results define a previously undiscovered requirement for STING signaling in the generation of peripheral Ag-specific CD4^+^ T cell tolerance.

### IFNAR expression is required for Ag-specific CNP-induced tolerance

Our published single-cell RNA sequencing data indicated that CNP treatment induces a strong type I IFN signature (*11*). In addition, type I IFNs have been reported to have an immunoregulatory function (*26*) and IFN-β therapy has had success in clinical treatment of certain autoimmune disorders (*20*, *21*). We thus sought to determine whether STING-induced type I IFNs may be required for Ag-specific CNP-induced tolerance. In line with the data presented in Fig. 5D and our published data (*11*), C57BL/6 BMDMs secreted IFN-β in a concentration-dependent manner upon CNP stimulation (fig. S5B), and this effect was independent of whether CNP contained Ag. To determine the functional role of type I IFNs in Ag-specific TIMP tolerance, we examined tolerance in mice lacking IFNAR expression. WT C57BL/6 and IFNAR^−/−^ mice were treated with CNP-OVA_323-339_ or CNP-MOG_35-55_ and primed with MOG_35-55_/CFA. The data show that IFNAR expression is required for Ag-specific CNP-induced tolerance (Fig. 7C).

Type I IFN receptors are ubiquitously expressed (*39*). In addition, there have been reports that intrinsic STING signaling within CD4^+^ T cells induces greater frequencies of T_reg_ cells (*19*, *26*). Thus, we sought to determine whether CD4^+^ T cell– and/or APC-intrinsic STING and IFNAR signaling was necessary for the activation/expansion of T_reg_ cells induced via Ag-specific CNP treatment. C57BL/6 2D2 MOG_35-55_-specific TCR transgenic CD90.1^+^ CD4^+^ T cells were sort purified and transferred into WT C57BL/6, STING^−/−^, or IFNAR^−/−^ recipient mice, which were then treated on Day 2 with either Unloaded CNP or CNP-MOG_35-55_. On Day 5, spleens were collected to enumerate and phenotype the 2D2 CD4^+^ T cells. CNP-MOG_35-55_ treatment significantly increased the total number of splenocytes in both WT and STING^−/−^ mice but not in IFNAR^−/−^ mice. Notably, CNP-MOG_35-55_ treatment significantly increased the number of FoxP3^+^ 2D2 CD4^+^ T cells and the number of 2D2 CD4^+^ T cells expressing the immunoregulatory molecules (CTLA-4, PD-1, and IL-10) only in WT C57BL/6 recipients and not in STING^−/−^ or IFNAR^−/−^ mice (Fig. 7D). Thus, Ag-specific CNP-induced tolerance requires both STING and IFNAR signaling within the APCs with little apparent role for CD4^+^ T cell–intrinsic STING and IFNAR signaling.

### Apoptotic cell–induced Ag-specific tolerance required both STING and IFNAR

**Fig. 8.**
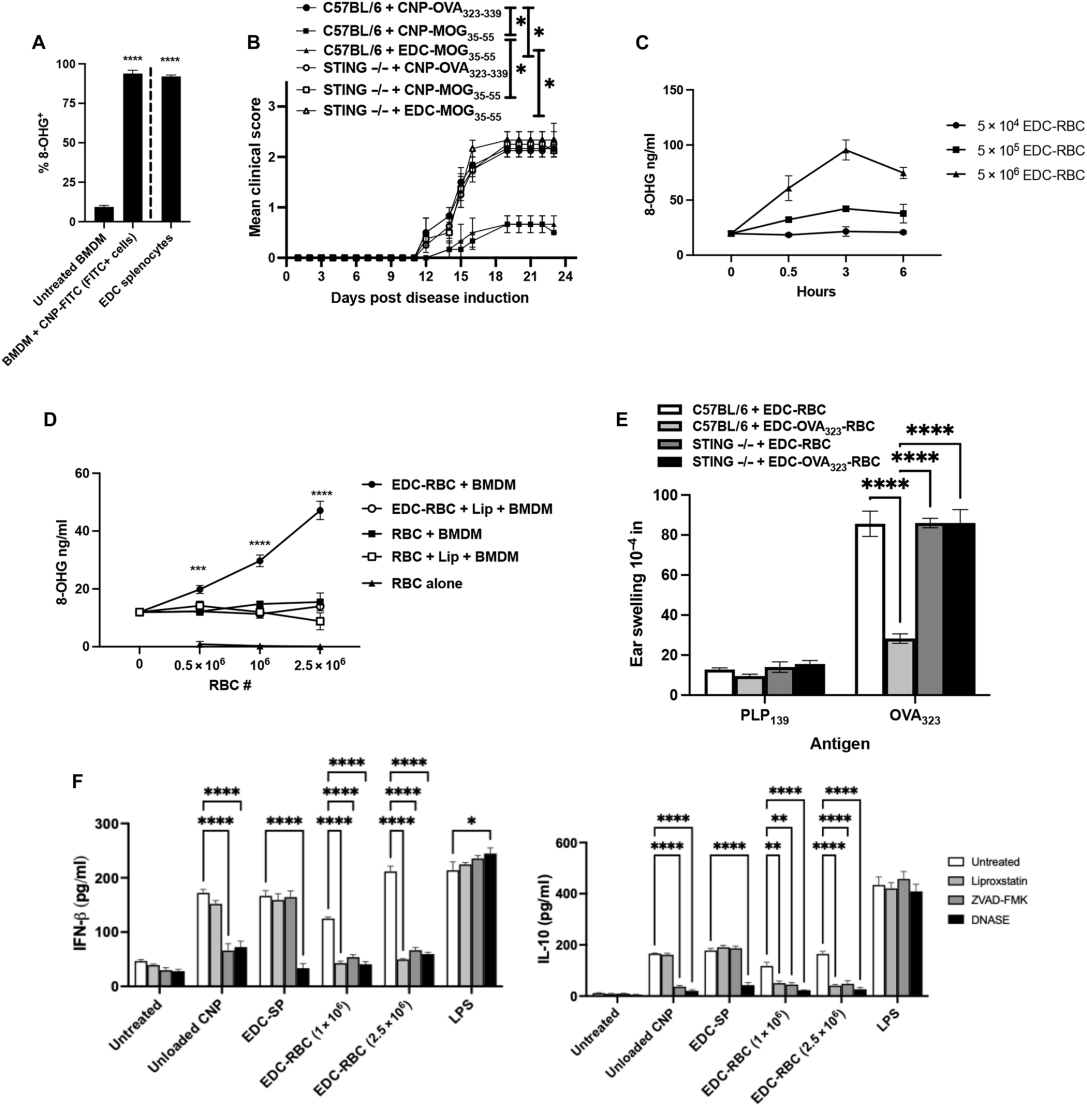
8-OHG is a common feature of apoptosis and is required for Ag-CNP–induced tolerance induction. BMDMs were cultured in the presence of CNP-FITC for 0 to 3 hours, and the frequency of CNP-FITC^+^, 8-OHG^+^, and cleaved Caspase-3^+^ cells was analyzed (**A**). C57BL/6 and STING^−/−^ mice (7- to 8-week-old female; *n* = 5 per treatment group) were tolerized intravenously with CNP-OVA, CNP-MOG_35-55_, or EDC-MOG_35-55_, primed subcutaneously with MOG_35-55_/CFA on Day 0, and followed for disease (**B**). BMDMs were cultured in the presence of increasing numbers of EDC-RBCs (5 × 10^4^ to 5 × 10^6^ RBCs), and the level of released 8-OHG was analyzed (**C**). BMDMs were cultured with increasing numbers of RBCs and EDC-RBCs +/− liproxstatin (Lip), and the level of released 8-OHG was analyzed at 3 hours (**D**). C57BL/6 and STING^−/−^ (7- to 8-week-old female; *n* = 4 per treatment group) were primed with OVA_323-339_/CFA, treated as indicated on Day 0, and DTH completed on Day 14 (**E**). BMDMs were cultured in the presence of Unloaded-CNP (5 μg/ml), EDC-SP (5 × 10^5^ cells), EDC-RBC (1 × 10^6^ and 2.5 × 10^6^ cells), or LPS +/− liproxstatin, ZVAD-FMK, and DNase I. Culture supernatants were collected at 20 hours, and the level of IFN-β and IL-10 was analyzed (**F**). One representative experiment of two is presented. Asterisks indicate a statistically significant difference as compared to the Control treatment group, *P < 0.05, **P < 0.01, ***P < 0.001, and ****P < 0.0001, respectively.

Prior studies by our laboratory used the intravenous infusion of MOG_35-55_ coupled to splenic leukocytes using EDC (MOG_35-55_-SP), a process that results in the apoptosis of the splenocytes (*4*), to induce tolerance in EAE. As phagocytosis of CNPs by BMDMs led to apoptosis (Fig. 5A), we thus asked whether EDC treatment of splenocytes results in 8-OHG expression similar to phagocytosis of CNPs and, consequently, whether 8-OHG positivity was a commonality among apoptotic cells. As a positive control, BMDMs were generated from C57BL/6 mice, and the cells were cultured in the presence of CNP-FITC. Alternatively, splenocytes from C57BL/6 mice were treated with EDC. Both FITC^+^ BMDMs and EDC-fixed splenocytes were 8-OHG^+^ (Fig. 8A). We next asked whether STING expression was required for Ag-SP–induced tolerance. Similar to CNP-MOG_35-55_, MOG_35-55_-SP treatment induced tolerance significantly decreased EAE disease severity in WT C57BL/6 mice but not in STING^−/−^ mice (Fig. 8B), thereby identifying STING as a common signaling pathway in Ag-SP and CNP tolerance.

We have previously shown that Ag-specific tolerance can be induced by EDC coupling of Ag to RBCs (Ag-RBC) (*40*, *41*). Large infusions of RBCs can lead to the induction of ferroptosis (*42*), which has previously been associated with downstream STING signaling in cancer (*43*). Thus, we sought to determine whether treatment with Ag-RBC induced 8-OHG. Coculture of EDC-treated RBCs induced 8-OHG release by BMDMs. In a cell number–dependent manner peaking at 3 hours (Fig. 8C). This differs from CNPs, which induce 8-OHG release peaking within the first 30 min of culture (Fig. 5C). As mature RBCs lack DNA, we next asked how EDC-coupled RBCs were capable of inducing 8-OHG. A prior report has indicated that large infusions of RBCs can induce ferroptosis and DNA damage within myeloid cells (*42*). Thus, we asked whether a large infusion of EDC-coupled RBCs induced 8-OHG and whether this was dependent on the presence of myeloid cells, EDC coupling of RBCs, and ferroptosis. We cocultured increasing numbers of RBCs that were either untreated or EDC fixed with or without BMDMs in the presence or absence of an inhibitor of ferroptosis liproxstatin-1 for 3 hours, and 8-OHG was measured in cell supernatants. Only in cocultures of BMDMs with EDC-treated RBCs did we see an increase in 8-OHG in an RBC number–dependent manner. Furthermore, liproxstatin treatment abrogated this increase in 8-OHG, showing that this increase in 8-OHG was through the ferroptosis pathway (Fig. 8D). To further determine whether the STING pathway was required for Ag-RBC–induced tolerance, WT C57BL/6 and STING^−/−^ mice were treated with EDC fixed-RBC (negative control) or OVA_323-339_-RBC, primed with OVA_323-339_/CFA, and OVA_323-339_-specific DTH determined on Day 14. As anticipated, OVA_323-339_-RBC treatment in WT C57BL/6 mice significantly decreased the OVA_323-339_-specific DTH response, but treatment failed to inhibit the OVA-specific DTH response in STING^−/−^ mice (Fig. 8E). Therefore, like Ag-specific CNP and Ag-SP treatment, Ag-RBC–induced tolerance also requires STING expression.

Next, we sought to further clarify the mechanism by which each method of Ag-specific tolerance induction functioned. BMDMs were cultured in the presence of Unloaded CNP, EDC-treated splenocytes (Ag-SP), EDC-treated RBCs (Ag-RBC), or LPS in the absence or presence of liproxstatin (an inhibitor of ferroptosis), ZVAD-FMK (a caspase-3 and apoptosis inhibitor), or DNase I (to degrade the released 8-OHG) (Fig. 8F). At 20 hours of culture, supernatants were collected, and the level of secreted IFN-β was quantified. DNase I inhibited the CNP-, Ag-SP–, and Ag-RBC–induced increase in IFN-β secretion. ZVAD-FMK inhibited the IFN-β release following culture in the presence of CNP and Ag-RBC but not by Ag-SP, which is consistent with the fact that Ag-SP are apoptotic shortly after fixation with EDC, which is before addition to the cultures (*41*). Last, liproxstatin, which inhibits ferroptosis, was able to only inhibit the Ag-RBC–induced increase in IFN-β secretion. Together, these data show that CNP-, Ag-SP–, and Ag-RBC–induced Ag-specific tolerance mechanistically function via a common downstream pathway beginning with the generation of 8-OHG and subsequent STING signaling.

**Fig. 9.**
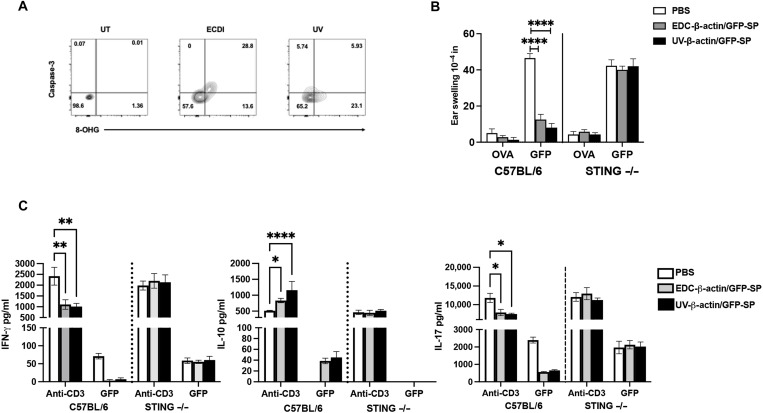
8-OHG generation is a common feature of apoptosis induction and is necessary for peripheral tolerance induction. C57BL/6 female splenocytes were treated with either EDC or 365-nm UV irradiation for 30 min, and the level of 8-OHG^+^ and cleaved Caspase-3^+^ was assessed via flow cytometry (**A**). C57BL/6 and STING^−/−^ (7- to 8-week-old female; *n* = 4 per treatment group) were primed with GFP/CFA and treated with either PBS, EDC–β-actin–GFP or UV–β-actin–GFP splenocytes (sp) on Day 0. DTH was completed on Day 14 postpriming. The data are presented as the means ± SEM (**B**). Splenocytes (5 × 10^5^ cells per well) were cultured ex vivo in the presence of anti-CD3 (1 μg/ml) or GFP (20 μg/ml), and culture supernatants were collected on Day 3 of culture. The levels of secreted IFN-γ, IL-17, and IL-10 were measured via Luminex (**C**). One representative experiment of two is presented. Asterisks indicate a statistically significant difference as indicated by the bars, *P < 0.05, **P < 0.01, and ****P < 0.0001, respectively.

Given that both CNP treatment and Ag-SP efficiently induce immune tolerance, we sought to clarify the mechanism(s) by which STING may also be a key regulator of self-tolerance induced following the clearance of apoptotic cells in vivo. Generation of 8-OHG appears to be a general feature of apoptosis, as indicated by the finding that ultraviolet (UV) irradiation of splenocytes leads to apoptosis as measured by cleaved caspase-3 and, concurrently, the induction of 8-OHG (Fig. 9A). Using splenocytes from β-actin–green fluorescent protein (GFP) mice, we induced apoptosis via UV irradiation and EDC treatment to determine whether STING expression is required for tolerance induced to a self-Ag, i.e., GFP, present within the transferred apoptotic β-actin–GFP splenocytes. Treatment with either EDC-treated or UV-irradiated β-actin–GFP splenocytes inhibited the GFP/CFA-induced DTH response in WT C57BL/6 mice but not in STING^−/−^ mice (Fig. 9B). Furthermore, ex vivo recall responses of lymph node cells from C57BL/6 mice, but not STING^−/−^ mice, that received either EDC-treated or UV-irradiated splenocytes secreted decreased levels of IFN-γ and IL-17 while enhancing the level of IL-10 secreted (Fig. 9C). Collectively, these findings demonstrate that the generation of 8-OHG is a by-product of apoptosis, which signals through STING to promote the generation of peripheral Ag-specific CD4^+^ T cell immune tolerance.

## DISCUSSION

Our laboratory has pioneered the development of various Ag-specific immune tolerance strategies. The most compelling finding was that intravenous delivery of Ag in the context of an apoptotic noninflammatory milieu is a highly efficient method of inducing immune tolerance to both self and foreign Ags. This finding was first observed by showing the tolerogenic capacity of Ag-coupled apoptotic splenocytes (Ag-SP) (*44*). Subsequent mechanistic studies showed that EDC treatment used for cross-linking Ag to the splenocyte surface by catalyzing peptide bond formation induced apoptosis of the splenocytes (*4*). Following intravenous injection, Ag-SP localize to the marginal zone of the spleen and the liver where the injected cells are taken up by macrophages and DCs (*4*). Subsequently, these APCs present the Ag that was coupled to the surface of apoptotic cells, and the couped Ag is presented by host MHC II molecules in the context of increased negative regulatory molecules and secreted IL-10 (*4*). The requirement of re-presentation of the Ag by host APCs, as opposed to direct Ag presentation by the Ag-coupled splenocytes, was shown by the ability of Ag-SP from MHC class II–deficient and MHC class I–deficient mice, as well as Ag-coupled RBCs to induce Ag-specific tolerance similarly to Ag-coupled syngeneic splenocytes (*40*, *41*).

We have shown that Ag-SP are highly effective for both prevention and treatment of mouse models and various autoimmune and allergic diseases, inducing both anergy and T_reg_ cell activation (*2*). On the basis of the efficacy of Ag-SP tolerance in mouse EAE models of MS, the aim of the first-in-man trial of Ag-coupled cells was to assess the feasibility, safety, and tolerability of a tolerization regimen in patients with MS that used a single infusion of autologous peripheral blood mononuclear cells EDC-coupled with seven HLA-DR2b-restricted myelin peptides derived from myelin oligodendrocyte glycoprotein (MOG), myelin basic protein (MBP), and myelin proteolipid protein (PLP) (MOG_1-20_, MOG_35-55_, MBP_13-32_, MBP_83-99_, MBP_111-129_, MBP_146-170_, and PLP_139-154_) (*5*). An open-label, single-center, dose-escalation study was performed in seven patients with relapsing-remitting and two patients with secondary progressive MS who were not on standard therapies. All patients had to show T cell reactivity against at least one of the myelin peptides used in the trial. Administration of Ag-coupled apoptotic autologous peripheral blood leukocytes (PBLs) had a favorable safety profile and was well tolerated. The highest dose of Ag-PBLs used in this study (3 × 10^9^) delivered an estimated 2.6 mg of total peptides and was found to significantly decrease the precursor frequency of peripheral blood myelin peptide-specific CD4^+^ T cells to four of the seven myelin peptides (*5*). Although this finding was very promising, the translational challenges associated with the cell-based approach and findings that polymeric biodegradable nanoparticles could be substituted for cells motivated the development of the CNP platform.

CNPs have been successfully applied to models of autoimmunity, allergy, and allogeneic transplant (*6*–*8*, *29*, *30*, *45*, *46*). Originally, intravenous injection of 500-nm-diameter “nonbiodegradable” carboxylated polystyrene (PS) particles coupled with peptides (Ag-PS) were shown to prevent the onset of disease in the mouse EAE model of MS (*6*). Our studies using PS nanoparticles identified that particles with diameters ranging from 500 to 1000 nm were most effective, and tolerance was dependent on particle uptake by scavenger receptors, including the macrophage receptor with collagenous structure (MARCO) (*47*, *48*). MARCO has been shown to be responsible for the uptake of PS beads that have a negatively charged surface (*47*). To make the platform more clinically translatable, Ag-bearing biodegradable nanoparticles developed from copolymers of lactic and glycolic acids (PLGA), Food and Drug Administration–approved for human use, was next tested. Initially, myelin Ags were coupled to the surface of PLGA nanoparticles, and later encapsulating Ags, were shown to induce Ag-specific tolerance for the prevention and treatment of EAE (*6*, *7*). Administration of Ag-coupled PLGA nanoparticles resulted in significantly reduced CNS infiltration of encephalitogenic T_H_1 (IFN-γ) and T_H_17 (IL-17a and GM-CSF) cells as well as inflammatory monocytes/macrophages. Tolerance was most effectively induced by intravenous infusion, with intraperitoneal and subcutaneous delivery having significantly reduced or no efficacy (*7*). The intravenous route has greater efficacy due to the direct delivery of Ag to the tolerogenic APC populations in the liver and spleen (*4*). CNPs engage the identical regulatory APCs in the liver and splenic marginal zone, which evolved to uptake and dispose of the huge numbers of apoptotic cells generated daily in the hematopoietic system while maintaining self-tolerance.

Here, we demonstrate for the first time to our knowledge that CNP-induced Ag-specific tolerance activates the expansion of Ag-specific T_reg_ cells, which is a highly active, multifaceted, and coordinated process. We also show that Ag-specific CNP-induced tolerance is a multistep mechanism and that the same tolerogenic pathways are used for both Ag-specific CNP-induced tolerance and tolerance induced via the presence of apoptotic cells within the spleen. After treatment with Ag-specific CNPs, CD4^+^ T cells specific for the cognate Ag contained within the CNP rapidly begin to proliferate within 3 days, as evident by both protein (Fig. 1) and transcript (Fig. 2) patterns of expression. For example, CNP-induced T_reg_ cells show a rapid up-regulation of immunoregulatory molecules such as CTLA-4, PD-1, and Lag-3, and blocking of any of these molecules abrogates immune tolerance in EAE (Figs. 1 and 3). We also show that treatment with Ag-specific CNP induces further expansion of the Ag-specific T_reg_ cells with each subsequent dose (fig. S3A). Functionality of T_reg_ cells is fundamentally linked to the Ag specificity of the T_reg_ cell (*49*) and the ability of the T_reg_ cells to attract immune effector cells to the site of Ag presentation (*50*). This makes functional sense as the Ag specificity would allow for spatially closer and increased frequency of interaction between the T_reg_ cell with effector CD4^+^ and CD8^+^ T cells. In support of the increased ability of the Ag-specific CNP-induced T_reg_ cells to attract effector immune cells, our data show that CCL3^+^ T_reg_ cells are increased following treatment (Fig. 1B). The further expansion of Ag-specific T_reg_ cells may also allow for the inhibition of effector CD4^+^ T cells of a different specificity via bystander suppression (fig. S3, B to D), in that repeat dosing of SJL/J mice with CNP-PLP_139-151_ was able to inhibit PLP_178-191_/CFA-induced EAE. However, the Ag-nonspecific T_reg_ cell function was only possible if the precursor frequency of the PLP_139-151_-specific CD4^+^ T cells was increased by PLP_139-151_-specific 5B6 CD4^+^ transgenic T cell transfer (fig. S3, E to G). However, this does not mean that Ag-specific CNP-induced regulation cannot modulate the response of T cells that are specific for tissue Ags not contained within the CNP. For example, the significant decrease in EAE disease severity in PLP_178-191_/CFA-primed mice treated with CNP-PLP_178-191_ on Day 12 inhibits the inflammatory response within the CNS, thereby inhibiting activation of spread epitope (PLP_139-151_)–specific CD4^+^ T cells both within the CNS and secondary lymphoid tissues (Fig. 4). Thus, if Ag-specific CNP treatment decreases tissue damage during an autoimmune response, the additional self-Ag is not presented by APCs to spread epitope–specific CD4^+^ T cells and, therefore, these CD4^+^ T cells of other specificities are not activated. The result is the induction of a tissue-specific tolerogenic response. The question of T_reg_ cell phenotype and stability is critical when considering T_reg_-dependent therapies. Although in vitro expanded or in vitro driven T_reg_ cells appear to be relatively unstable (*51*), our published and present findings suggest that Ag-specific CNP treatment induces long-lived, stable Ag-specific T_reg_ cell populations (Fig. 2), and further study of this mechanism is ongoing.

It is noteworthy that the molecular mechanisms underlying both Ag-apoptotic cell tolerance and Ag-specific CNP tolerance rely on the activity of the STING pathway (Fig. 7). This finding is relevant for the clearance of apoptotic cells from the body, and the present findings support the published data showing that STING activity regulates IL-10 production within the gut (*52*). STING recognizes DNA within the cytosol of cells to coordinate an immune response. Treatment of splenocytes with EDC or phagocytosis of CNPs by BMDMs, RAW cells (*11*), or splenocytes was sufficient to induce oxidized DNA, which is a well-documented ligand crucial for cGAS/STING signaling (*36*). Regarding the wider implications of the present findings, 8-OHG was increased following the induction of apoptosis via UV irradiation (Fig. 9). These findings suggest that the generation of oxidized DNA is a common feature of apoptosis and would appear to complicate the prior assumptions that apoptosis is an immunologically silent form of cell death. Instead, the clearance of apoptotic cells appears to actively induce regulatory phenotypes within the associated APCs and T cells. Of particular interest to the present findings, the myeloid cells that initially undergo apoptosis after CNP uptake do not appear to be the APCs presenting Ag to the CD4^+^ T cells. Alternatively, there appears to be a secondary population of APCs that “ingests” the apoptotic cell/8-OHG/Ag to deliver a tolerogenic signal to T cells. On the basis of the reported ability of pDCs to secrete significate levels of type I IFNs (*53*) and the increased number of these cells post–Ag-specific CNP treatment (Fig. 1 and fig. S1), we hypothesize that these cells may be the local source of type I IFN post–CNP dosing. The present data also show that Ag-specific CNP dosing results in a significant increase in the number of PD-L1^+^ cDC2s (Figs. 1F and 6E and figs. S1E and S5E), a population of APCs known to activate CD4^+^ T cells and been shown to have tolerogenic function (*54*, *55*).

Because the presence of oxidized DNA activates the STING/IFNAR pathway that has been clearly shown to be required for Ag-specific tolerance induction following the transfer of apoptotic cells in vivo (Fig. 8, B and E), the question of how the STING/IFNAR pathway can be both inflammatory, e.g., in the setting of antiviral or antitumor immune responses, and tolerogenic arises. Although specific details remain to be identified, the difference may depend on the duration of the STING/IFNAR pathway activation, the location of the stimulation, i.e., in the peripheral lymph nodes versus the spleen or liver, and the presence or absence of TLR ligands or other alarmins. This scenario would also appear to be multifactorial as cotreatment of mice with LPS was not sufficient to inhibit the induction of Ag-specific tolerance (fig. S4D).

The efficacy of the CNP tolerance induction platform for the treatment of a variety of autoimmune and allergic indications in mouse disease models (*6*–*8*, *29*, *30*, *45*, *46*), and the initial efficacy in a phase 1/2a clinical trial in celiac disease (*9*), is likely due to the fact that it activates a molecular pathway which evolved in the immune system to maintain tolerance to self-Ags liberated by apoptotic cell death of hematopoietic cells. The fundamental linkage between apoptotic cell clearance and the induction of Ag-specific tolerance has been well documented (*56*). The present findings show that CNP-induced myeloid cell apoptosis is linked to Ag-specific CNP-induced tolerance, which is supported by various previously published findings. Arterial blood entering the spleen passes through the splenic marginal zone that contains reticular fiber–associated marginal zone macrophages (*57*, *58*). As previously discussed, these marginal zone macrophages express MARCO and are required for Ag-specific CNP-induced tolerance (*6*). Further studies have shown that disruption of the marginal zone macrophages results in a loss of systemic tolerance to dsDNA and an increase in mortality over time (*59*). In addition, in the absence of marginal zone macrophages, transferred apoptotic cells localize within the PALS, where these cells are phagocytosed by CD68^+^ F4/80^+^ macrophages that up-regulate MHC II and CD86. Although the loss of marginal zone macrophages, MARCO expression, or STING expression alone by mice on the C57BL/6 background has not been associated with an increased incidence of autoimmune disease, as noted above, the continual low dosing of mice with disrupted marginal zone macrophages results in a significant increase in anti-dsDNA and mortality over time (*59*). Together, these findings point to a major role for the MARCO/STING/IFNAR pathways in the maintenance of self-tolerance. However, the findings also suggest that redundant pathways are present to help maintain self-tolerance. In sum, Ag-specific CNPs can be characterized as “surrogates” of apoptotic bodies, i.e., similar in size (400 to 600 nM) and negative charge, which direct their Ag cargo to MARCO-expressing APCs that undergo apoptosis following CNP phagocytosis. Consequently, uptake of apoptotic debris in the context of 8-OHG and Ag-induced up-regulation of the expression of protolerogenic regulatory receptors and cytokines skew the microenvironment to favor differentiation of Ag-specific FoxP3^+^ T_reg_ and Tr1 regulatory cells.

## MATERIALS AND METHODS

### Materials and manufacture of CNP nanoparticles

Acid-terminated PLGA polymer was purchased from Lactel Absorbable Polymers (Evonik Corporation, AL). Ovalbumin (OVA) was purchased from Sigma-Aldrich (St. Louis, MO), and GFP was from MyBioSource (Vancouver, BC, Canada). Peptides (PLP_139-151_, PLP_178-191_, MOG_35-55_, and OVA_323-339_) were purchased from Genemed Synthesis (Torrance, CA). For CNPs encapsulating peptides or proteins, PLGA solution was mixed with each recombinant protein to generate a water-in-oil emulsion. This was mixed with a proprietary blend of surfactants and organic solvents to form an oil-in-water secondary emulsion. The solvent was removed by evaporation, yielding PLGA nanoparticles that encapsulated the protein/peptide, which were washed, filtered, and concentrated via tangential flow filtration. CNPs were supplied as a lyophilized powder containing ~100 mg of PLGA nanoparticles per vial with average nanoparticle diameters of 400 to 800 nm and zeta potentials of −30 to −60 mV. The protein content was ≥3 μg of recombinant protein per milligram of PLGA.

### Mice

Six- to 8-week-old SJL/J and C57BL/6 mice were purchased from Envigo and allowed to acclimate to the Northwestern University animal care facility for 1 week before use. 5B6 TCR transgenic (PLP_139-151_-specific)–Thy1.1^+^, 2D2 TCR transgenic (MOG_35-55_-specific)–Thy1.1^+^, STING^−/−^, IFNAR^−/−^ and, β-actin–GFP mice were bred and housed in a specific pathogen–free environment in the Northwestern University Center for Comparative Medicine. Northwestern University has an Animal Welfare Assurance on file with the Office of Laboratory Animal Welfare (A3283-01). Northwestern University conducts its reviews in accordance with US Public Health Service (USPHS) regulations and applicable federal and local laws. The composition of the Northwestern IACUC meets the requirements of the USPHS policy and the Animal Welfare Act Regulations. The animal approval for this project is IS00002901_IM36.

### TCR transgenic T cell transfer, antigen/CFA priming, and EAE disease scoring

WT mice received 3 × 10^6^ to 5 × 10^6^ Thy1.1^+^ 5B6 or 2D2 CD4^+^ T cells or vehicle [phosphate-buffered saline (PBS)] via intravenous tail vein injection. On the indicated days post–T cell transfer, recipient mice were treated intravenously with CNPs encapsulating antigen (PLP_139-151_, PLP_178-191_, MOG_35-55_, or OVA_323-339_) or unloaded CNPs (2.5 mg per dose). For EAE induction (Day 0), mice were shaved (~1 inch–by–1 inch square on the back between the hind flanks) and injected subcutaneously with 100 μl of an emulsion containing 200 μg of *Mycobacterium tuberculosis* H37Ra (BD Biosciences; San Jose, CA) in CFA and 50 μg of PLP_139-151_, 100 μg of PLP_178-191_, 200 μg of MOG_35-55_, 100 μg OVA_323-339_, 100 μg OVA, or 100 μg of GFP distributed over three sites. For induction of MOG_35-55_ EAE, mice also received 200 ng of *Bordetella pertussis toxin* (PTx) in 200 μl of PBS intraperitoneally on days 0 and 2 postpriming. For the inactivation of T_reg_ cells, mice were treated with an isotype- and species-matched control Ab or anti-CD25 (Clone _PC-61.5.3, 500 μg per dose in 200 μl of 1x Dulbecco’s phosphate-buffered saline (DPBS) via intraperitoneal injection) on Days 5 and 7 postpriming. For the blockade of regulatory proteins, mice were with an isotype- and species-matched control Ab, anti–CTLA-4, anti–PD-1, anti–TGF-β, or anti–IL-10 (100 μg per dose in 200 μl of 1x DPBS via intraperitoneal injection) on Days 0, 2, 4, 7, 9, and 11 post–disease induction. Individual animals were observed at the indicated time points, and clinical scores were assessed in a blinded manner on a 0 to 5 scale: 0, no abnormality; 1, limp tail; 2, limp tail and hind limb weakness; 3, hind limb paralysis; 4, hind limb paralysis and forelimb weakness; and 5, moribund. For the blockade of 8-OHG and DTH, mice were injected with either 250 μg of anti–8-OHG (Genetex) or 250 μg of IgG2b on Day −3. Mice were then primed with OVA and treated with either unloaded-TIMP or TIMP-OVA on Day 0.

### Antigen-coupled splenocyte/RBC tolerance

Peripheral tolerance induction using peptide-coupled splenocytes was performed as previously described (*41*). Spleens were removed from C57BL/6 female mice, RBCs were lysed, and leukocyte suspensions were incubated with EDC (150 mg/3.2 × 10^8^ cells; Sigma-Aldrich) and peptide (1 mg/ml) on ice, with shaking for 1 hour. The coupled cells (Ag-SP) were washed 3x in PBS, centrifuged, and filtered to remove cell clumps. Ag-SP was resuspended at 2.5 × 10^8^/ml in PBS. Each mouse received 5 × 10^7^ Ag-SP in 200 μl of PBS given by intravenous injection 7 days before disease induction in EAE or on the day of antigen priming in DTH. For Ag-RBC tolerance (*40*), RBCs were collected via eye bleed, and EDC coupled with peptide identically to the Ag-SP. Tolerance was induced by intravenous injection of 1 × 10^9^ Ag-RBC in 200 μl of PBS per mouse. Coupling efficiency has previously been determined to be ~30%, yielding 24 to 55 μg of peptide/5 × 10^7^ splenocytes (*41*) . For tolerance to GFP splenocytes from β-actin, GFP mice were processed as above and then treated to either UV irradiation for 1 hour or EDC treatment without Ag as described above. Cells were then injected intravenously at the same concentration as previously described above in EDC tolerance.

### DTH and ex vivo recall cultures

On Day 14 postpriming, mice were assayed for DTH. Mice were anesthetized via inhalation with isoflurane, and the thickness of both ears was measured using a dial thickness gauge. Negative control protein/peptide and specific protein/peptide (10 μl at 1 mg/ml) in PBS as listed in each figure was injected into the left and right ear, respectively. The increase in ear thickness was determined after 24 hours. On indicated days post–Ag/CFA priming or induction of EAE, anesthetized mice were euthanized, spleens and draining lymph nodes were collected, and single-cell suspensions were prepared by mashing the tissues through 100-μm Nitex filters and then washing the filters with 10 ml of Hanks’ balanced salt solution (HBSS) + 5% fetal calf serum (FCS). RBCs from the spleen were lysed via tris-NH_4_Cl treatment for 4 min at 37°C. Cells were pelleted, washed 3x with HBSS + 5% FCS, strained through a 40-μm Nitex filter to remove cellular debris, and counted on a hemocytometer with trypan blue exclusion. For in vitro reactivation cultures, 1 × 10^6^ total splenocytes or lymph node cells were plated in replicate wells on a 96-well flat-bottom plates with complete RPMI medium plus anti-CD3 (1 μg/ml), OVA_323-339_, MBP_84-104_, PLP_139-151_, or PLP_178-191_, and whole OVA or MBP (20 μg/ml). Culture supernatants were collected at 72 hours post–culture initiation, and the levels of secreted cytokines were determined via Luminex using a Mouse Cytokine/Chemokine Magnetic Bead Panel (Millipore).

### Flow cytometry

Single-cell suspensions were prepared from spleens and lymph nodes and RBCs lysed using tris-NH_4_Cl. For the CNS, single-cell suspensions were prepared by mincing the tissue in 2 ml of Accutase (MilliPore), followed by incubation at 37°C for 30 min. Following the enzyme digestion, the CNS samples were disrupted using a 100-μm cell strainer and rinsed 2x with 10 ml of HBSS + 5% FCS, and the cells were pelleted. For intracellular staining, 1 × 10^6^ cells in 200 μl of complete RPMI medium were cultured in the presence of phorbol 12-myristate 13-acetate (PMA; 50 ng/ml), ionomycin (500 or 200 ng/ml) for 2 hours, and brefeldin A (10 μg/ml) for an additional 2 hours. Cells were collected, and Live/Dead stain was performed per the manufacturer’s instructions. The cells were then incubated in Flow Cytometry Staining Buffer (1x PBS + 5% FCS) plus Fc Block (Anti-Mouse CD16/CD32 Purified; Thermo Fisher Scientific) in the dark at 4°C for 30 min. Samples were washed 3x with 1x PBS + 5% FCS and then incubated with antibodies for cell surface staining (diluted 1:200 in 1x PBS + 5% FCS) in the dark at 4°C for 30 min. For intracellular staining, Fixation/Permeabilization (Intracellular Fixation & Permeabilization Buffer Set; Thermo Fisher Scientific) working solution was added to each tube and pulse vortexed. Samples were incubated in the dark at 4°C for 60 min, washed 3x with 1x Permeabilization Buffer, and blocked with 2% normal rat serum at room temperature for 15 min. Fluorochrome-conjugated antibodies (diluted 1:200 in 1x Permeabilization Buffer) for detection of intracellular Ag(s) were added to the cells, and samples were incubated in the dark at room temperature for 30 min and then washed 3x with 1x Permeabilization Buffer, followed by three washes with 1x PBS. Stained cells were resuspended in 1x PBS + 5% FCS, samples were analyzed on a BD Celesta Flow Cytometer (BD Bioscience), and the data analyzed using FloJo Version 9.5.2 software (Tree Star Inc.; Ashland, OR). The specific antibodies used are listed in table S1.

### RNA sequencing

5B6 CD4^+^ T cells were adoptively transferred to WT SJL/J mice on Day 0, and mice were treated on Day 3 with either unloaded or CNP-PLP_139-151_. On Day 6, mice were euthanized and the 5B6 CD4^+^ T cells were sort purified and RNA was isolated using the RNeasy Pure mRNA bead kit (QIAGEN, Hilden, Germany) according to the manufacturer’s instructions. cDNA libraries were then constructed for sequencing using TruSeq mRNA-Seq Library Prep. Libraries were then read on an Illumina Hiseq.

For computational analysis, data were analyzed by ROSALIND (https://rosalind.bio/), with a HyperScale architecture (ROSALIND Inc., San Diego, CA). Reads were trimmed using cutadapt1. Quality scores were assessed using FastQC2. Reads were aligned to the *Mus musculus* genome build mm10 using STAR3. Individual sample reads were quantified using HTseq4 and normalized via Relative Log Expression (RLE) using DESeq2 R library5. Read Distribution percentages, violin plots, identity heatmaps, and sample MDS (multidimensional scaling) plots were generated as part of the QC step using RSeQC6. DEseq2 was also used to calculate fold changes and *P* values and perform optional covariate correction. Clustering of genes for the final heatmap of differentially expressed genes was done using the PAM (Partitioning Around Medoids) method using the fpc R library7. For generation of the plots, ggplot2 was used to generate a volcano plot and heatmap was generated using Complex Heatmap (2.14). For the enrichment analysis, the gseGO function was used followed by dotplot for visualization all from the clusterProfiler package. All data visualization and enrichment analysis was performed in R ver 4.2.3.

### BMDM and splenocyte cultures

Bone marrow–derived myeloid cells were generated as previously described (*11*). BMDMs from C57BL/6 and STING^−/−^ mice were then cultured with various combinations of unloaded CNPs (5 μg/ml), DNase I (2 μg/ml; Promega), and LPS (1 μg/ml; Sigma-Aldrich) for 24 hours. Supernatants were then harvested, and IL-10 and IFN-β were detected using Luminex (Millipore) and ELISA (Abcam), respectively. BMDMs were cultured with combinations of either unloaded-CNPs or CNP-FITC +/− DNase I (5 μg/ml) or 5 × 10^5^ EDC-fixed splenocytes. Supernatants were harvested for quantification of 8-OHG via ELISA (Abcam) according to the manufacturer’s instructions. Splenocytes from 5B6, C57BL/6, and STING^−/−^mice were processed into single-cell suspensions as described above. Cells were then resuspended at 2.5 × 10^6^/ml in 200 μl of complete RPMI 1640 with increasing concentrations of the STING agonist, G3-YSD (Invivogen) and PLP_139-151_ (20 μg/ml) for 72 hours in 96-well plates. For experiments with culture with CNP-OVA_323_ splenocytes from C57BL/6, STING^−/−^, and IFNAR^−/−^, mice were processed as described as above and cocultured with CNP-OVA_323-339_ (5 μg/ml) for 24 hours.

### Statistical analyses

All statistical analyses were performed using GraphPad Prism software. Unpaired Student’s *t* test was used for comparison of two groups, one-way analysis of variance (ANOVA) was used for comparison of more than two groups, and two-way ANOVA was used for comparison of more than two groups and two variables. Values are represented as means ± SEM. Statistical significance is denoted by the annotation: *P < 0.05, **P < 0.01, ***P < 0.001, and ****P < 0.0001.
