# Supplementary material for: STING/type I interferon pathway is required for antigen-containing PLGA nanoparticle- and apoptotic cell–induced CD4+ T cell tolerance
Source: Sci Adv. 2026 Jan 2;12(1):eadv8860. doi: 10.1126/sciadv.adv8860 (PMC12758558; doi:10.1126/sciadv.adv8860)
Supplement: Supplementary file 1 — Figs. S1 to S5 Table S1 [file sciadv.adv8860_sm.pdf]

Science Advances
AAAS

Supplementary Materials for

STING/type I interferon pathway is required for antigen-containing PLGA nanoparticle- and apoptotic cell–induced CD4+ T cell tolerance

Joseph R. Podojil *et al.*

Corresponding author: Stephen D. Miller, s-d-miller@northwestern.edu

Sci. Adv. **12**, eadv8860 (2026)
DOI: 10.1126/sciadv.adv8860

This PDF file includes:

Figs. S1 to S5
Table S1

Supplemental Figure 1

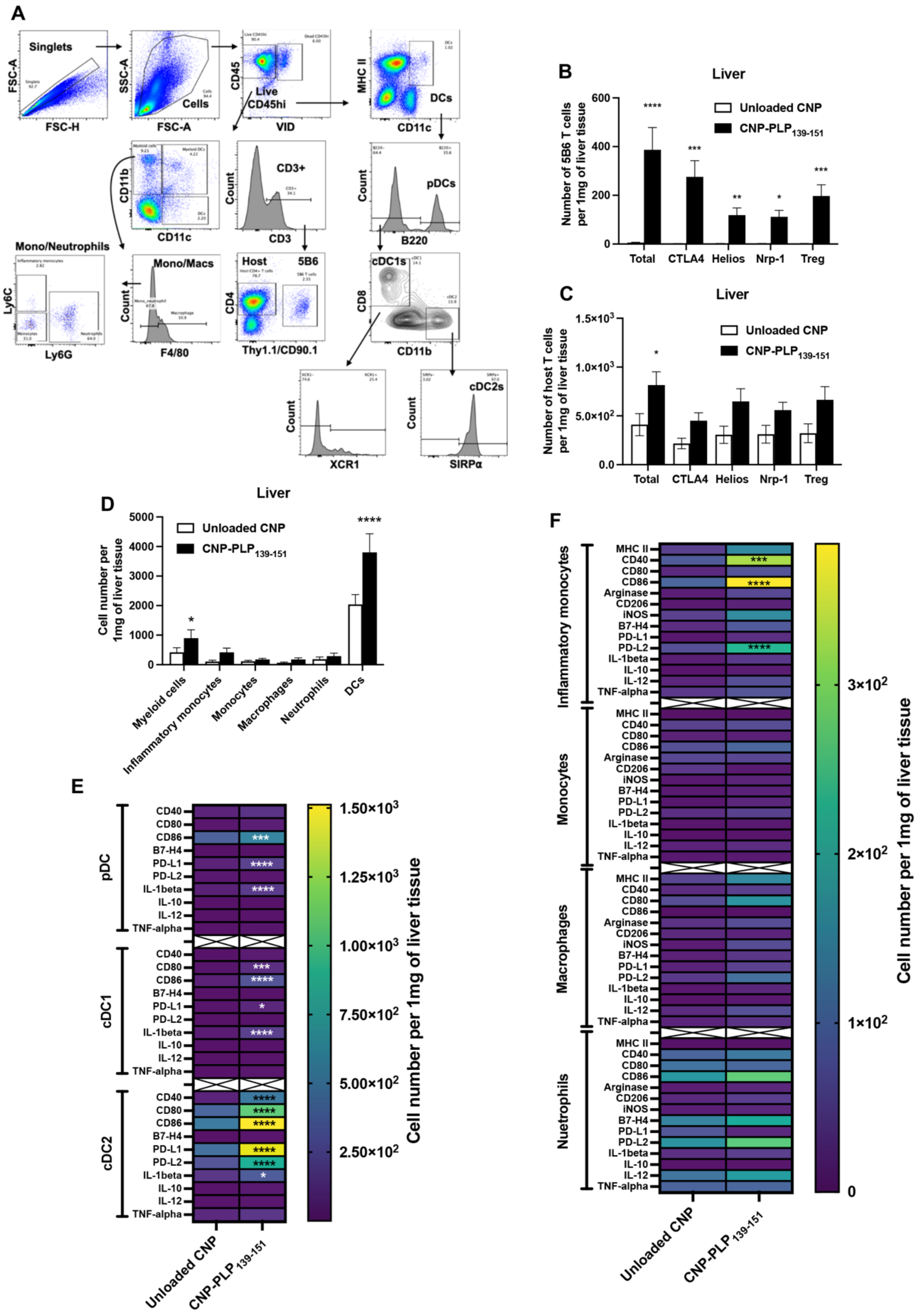

Supplemental Figure 1. CNP-PLP$_{139-151}$ treatment increases the number of Ag-specific Treg cells within the liver. The total cell gate was plotted for CD45^{hi} *versus* VID stain negative for the liver CD45^{hi} leukocytes. This live leukocyte population was then gated on CD3^{+} cells in the CD4^{+} T cell panels or CD11b *versus* CD11c for the APC panels. To identify the host *versus* transferred 5B6 CD90.1^{+} CD4^{+} T cells or 2D2 CD90.1^{+} CD4^{+} T cells, the CD4^{+} CD90.1^{-} T cells are the host CD4^{+} T cells and the CD4^{+} CD90.1^{+} T cells are the transferred 5B6 or 2D2 CD4^{+} T cells. To phenotype pDCs, cDC1s, cDC2s, inflammatory monocytes, macrophages, monocytes, and neutrophils the presented markers were used. The final immune cell populations were then phenotyped further for specific activation and regulatory markers (**A**). SJL/J (CD90.2^{+}) mice (7–8-week-old female; n=4 per treatment group) received 3x10^{6} sort 5B6 CD90.1^{+} CD4^{+} T cells via i.v. injection on Day 0. On Day 2, mice were treated with either Unloaded CNP or CNP-PLP$_{139-151}$ (2.5mg/dose) via i.v. injection, livers were collected on Day 7, and cells analyzed by flow cytometry for the phenotype of 5B6 CD4^{+} T cells (**B**), and the number of host CD4^{+} T cells that expressed regulatory markers (**C**). The phenotype and number of pDCs, cDC1s, cDC2s, inflammatory monocytes, macrophages, monocytes, and neutrophils was assessed by flow cytometry. The number of number of cells within each population is presented (**D**), as are the number of DCs (**E**) and myeloid cells (**F**) expressing the respective markers. The data are presented as the mean number of cells expressing the respective phenotype $\pm$ S.E.M. One representative experiment of three is presented. Asterisks indicate a statistically significant difference as compared to the Unloaded TIMP treatment group, *$p < 0.05$, **$p < 0.01$, ***$p < 0.001$, and ****$p < 0.0001$, respectively.

Supplemental Figure 2

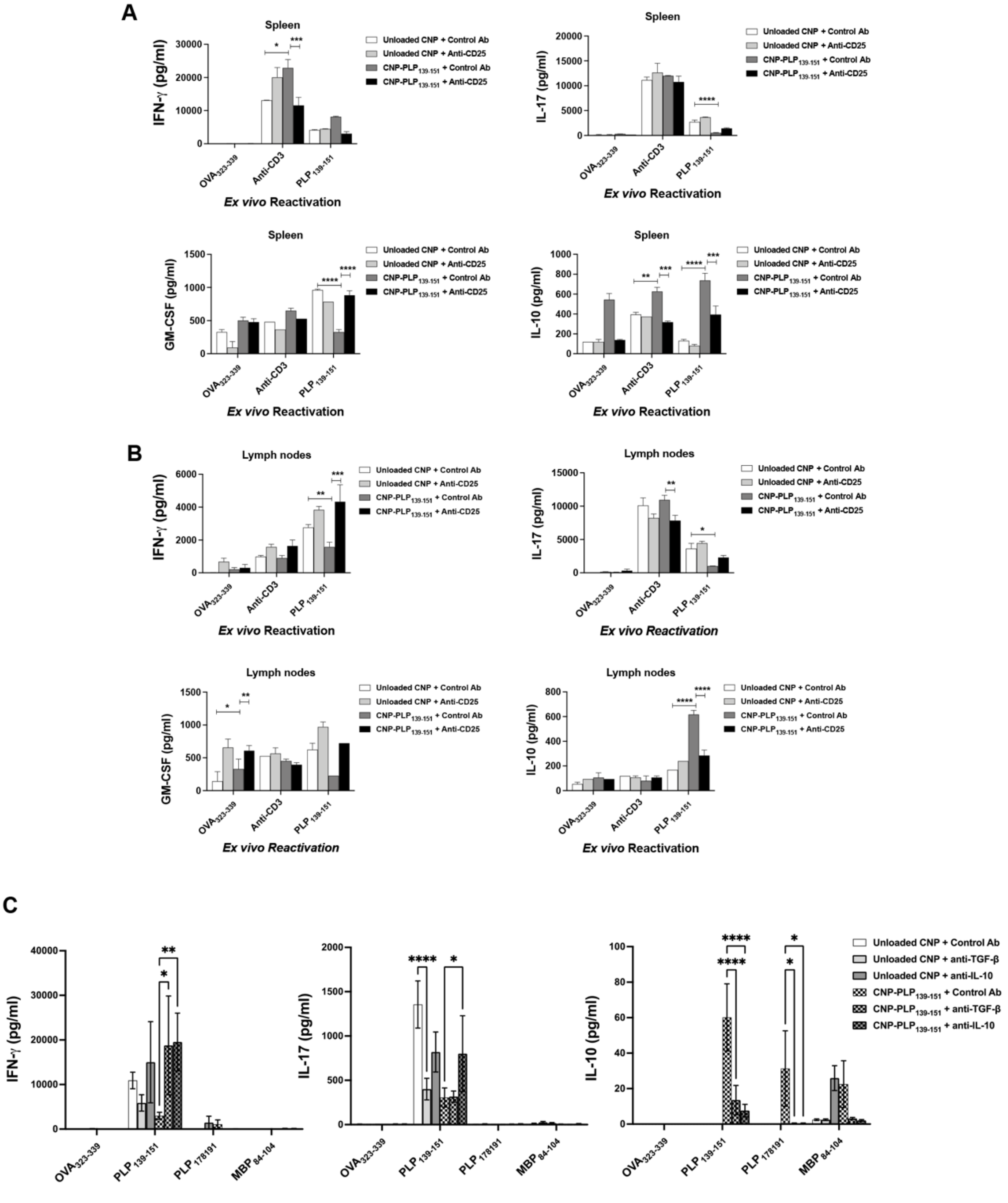

Supplemental Figure 2. Functional Treg cells, TGF-β, and IL-10 are required for the CNP-PLP$_{139-151}$-induced alterations in *ex vivo* recall responses. For the mice presented in the disease course shown in **Figure 3A**, *ex vivo* recall responses of the spleen and inguinal lymph nodes was completed. Total splenocytes (**A**) and inguinal lymph node cells (**B**) ($5x10^5$ cells/well) were cultured in the presence of anti-CD3 (1 μg/ml), OVA$_{323-339}$, or PLP$_{139-151}$ (20 μg/ml) and culture supernatants were collected on Day 3 of culture. The level of secreted IFN-γ, IL-17, GM-CSF, and IL-10 were measure via Luminex. For the mice presented in the disease course shown in **Figure 3C** *ex vivo* recall responses of the spleen were completed. Total splenocytes ($5x10^5$ cells/well) were cultured in the presence of anti-CD3 (1 μg/ml), OVA$_{323-339}$, PLP$_{139-151}$, or PLP$_{178-191}$, MBP$_{84-104}$ (20 μg/ml) and culture supernatants were collected on Day 3 of culture. The level of secreted IFN-γ, IL-17, and IL-10 were measure via Luminex (**C**). The data are presented as the mean concentration of cytokine per ml of culture supernatant +/- S.E.M. One representative experiment of two is presented. Asterisks indicate a statistically significant difference as indicated by the bars * p <0.05, **p < 0.01, ***p < 0.001, and ****p < 0.0001, respectively.

Supplemental Figure 3

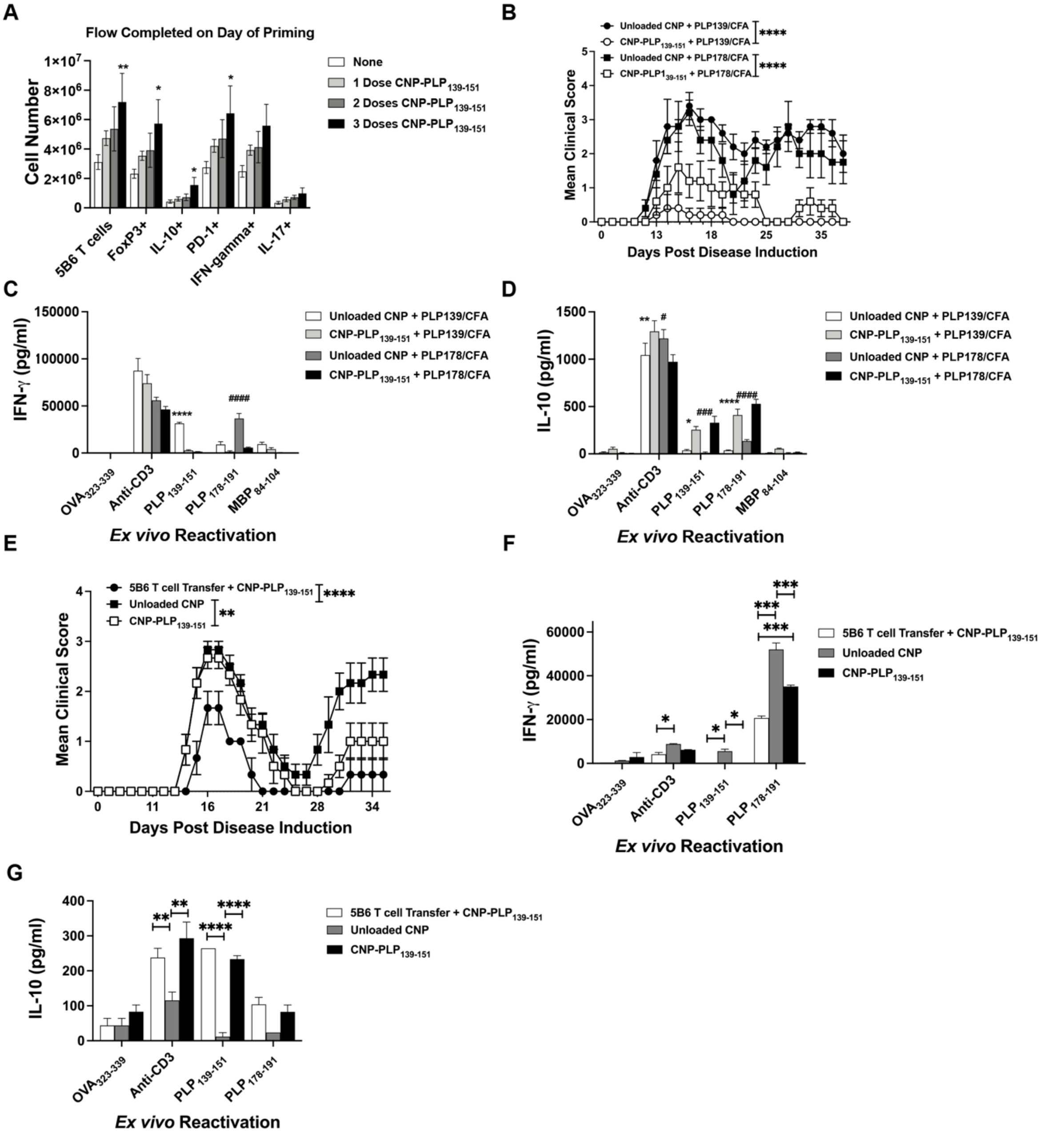

Supplemental Figure 3. Multiple CNP-PLP$_{139-151}$ treatments increase the number of 5B6 T cells such that bystander suppression of PLP$_{178-191}$/CFA-induced EAE is induced. SJL/J (CD90.2$^+$) mice (7–8-week-old female; n=5 per treatment group) received 3x10^6 sorted 5B6 CD90.1$^+$ CD4$^+$ T cells via i.v. injection on Day 0. On Days 2, 9, and 16, mice were treated with either Unloaded CNP or CNP-PLP$_{139-151}$ (2.5 mg/dose) via i.v. injection, spleens were collected on Day 22, and splenocytes analyzed by flow cytometry to determine the phenotype of 5B6 CD4$^+$ T cells. The data are presented as the mean number of 5B6 CD4$^+$ T cells expressing each marker **(A)**. On Day 22, a separate cohort of mice that had received 5B6 CD4$^+$ T cells on Day 0 and three doses of either Unloaded CNP or CNP-PLP$_{139-151}$ were primed with either PLP$_{139-151}$/CFA or PLP$_{178-191}$/CFA (Day 0 of the disease course). The mice were followed for disease severity and the data are presented as the mean clinical score over time (**B**). On Day 38 of the disease course, spleens were collected, and total splenocytes (5x10^5 cells/well) were cultured in the presence of anti-CD3 (1 μg/ml), OVA$_{323-339}$, PLP$_{139-151}$, PLP$_{178-191}$, or MBP$_{84-104}$ (20 μg/ml). Culture supernatants were collected on Day 3 of culture to measure the level of secreted IFN-γ (**C**) and IL-10 (**D**). As a positive control for the CNP-PLP$_{139-151}$ treatment-induced decrease in PLP$_{178-191}$/CFA-induced EAE, one group of mice received 5B6 CD4$^+$ T cells (3x10^6 cells) on Day -2 prior to the first CNP-PLP$_{139-151}$ treatment. The other two treatment groups did not receive 5B6 CD4$^+$ T cells and only received either Unloaded CNP or CNP-PLP$_{139-151}$ (2.5 mg/dose) on Days 2, 9, and 16. On Day 22 (Day 0 of the disease course), mice were primed with PLP$_{178-191}$/CFA, and mice were followed for disease. The data are presented as the mean clinical score over time (**E**). On Day 35 of the disease course, spleens were collected, and total splenocytes (5x10^5 cells/well) were cultured in the presence of anti-CD3 (1μg/ml), OVA$_{323-339}$, PLP$_{139-151}$, or PLP$_{178-191}$ (20 μg/ml). Culture supernatants were collected on Day 3 of culture to measure the level of secreted IFN-γ (**F**) and IL-10 (**G**) via Luminex. The data are presented as the mean concentration of cytokine per ml of culture supernatant $\pm$ S.E.M. One representative experiment of two is presented. Asterisks indicate a statistically significant difference as indicated by the bars *p <0.05, **p < 0.01, ***p < 0.001, and ****p < 0.0001, respectively.

Supplemental Figure 4

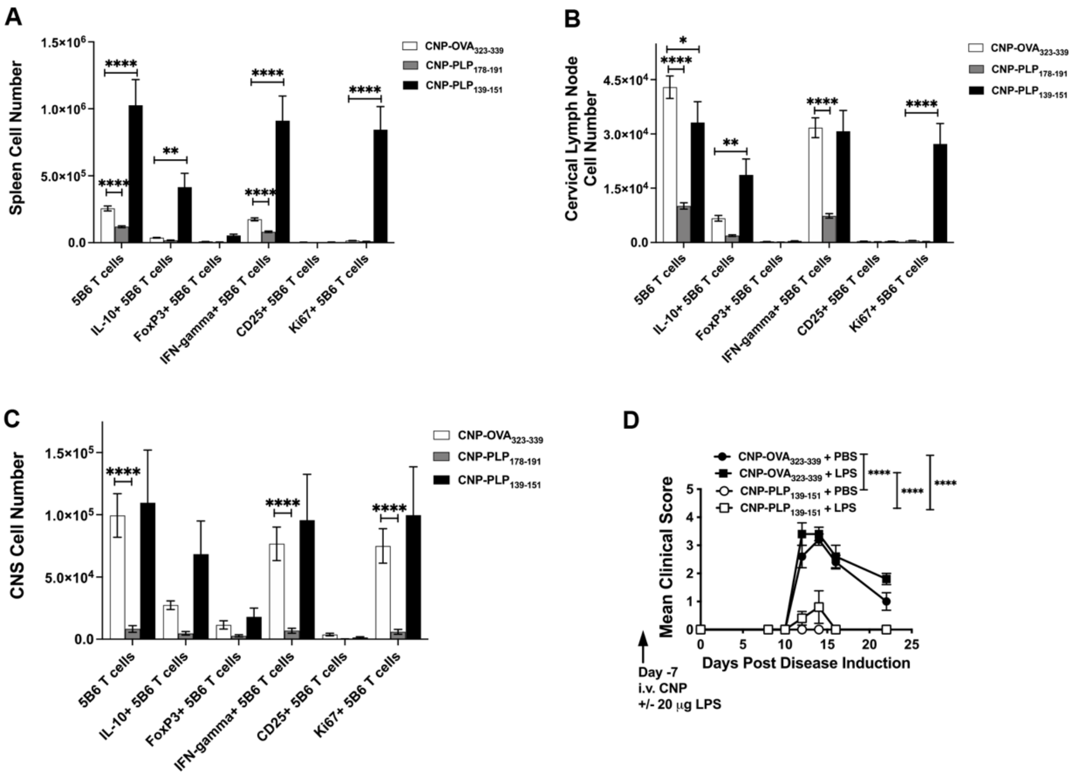

Supplemental Figure 4. Treatment of mice with CNP-PLP$_{139-151}$ *versus* CNP-PLP$_{178-191}$ during PLP$_{178-191}$/CFA-induced EAE differentially alters 5B6 CD4$^+$ T cells. For the mice presented in the **Figure 5C-E** the mean numbers of 5B6 CD4$^+$ T cells expressing each respective marker is presented for the spleen (**A**), the cervical lymph nodes (**B**), and the CNS (**C**). The data are expressed as the mean number of cells expressing the respective phenotype $\pm$ S.E.M. One representative experiment of two is presented. On Day -7 SJL/J mice (7–8-week-old female; n=5 per treatment group) treated i.v. with either CNP-OVA$_{323-339}$ or PLP$_{139-151}$ (2.5 mg/dose) in the absence or presence of LPS (20 mg/dose). On Day 0 mice were primed with PLP$_{139-151}$/CFA s.c. The mice were followed for disease, and the data are presented as the mean clinical score +/- S.E.M. over time (**D**). One representative experiment of two is presented. Asterisks indicate a statistically significant difference as indicated by the bars *p <0.05, **p < 0.01, ***p < 0.001, and ****p < 0.0001, respectively.

Supplemental Figure 5

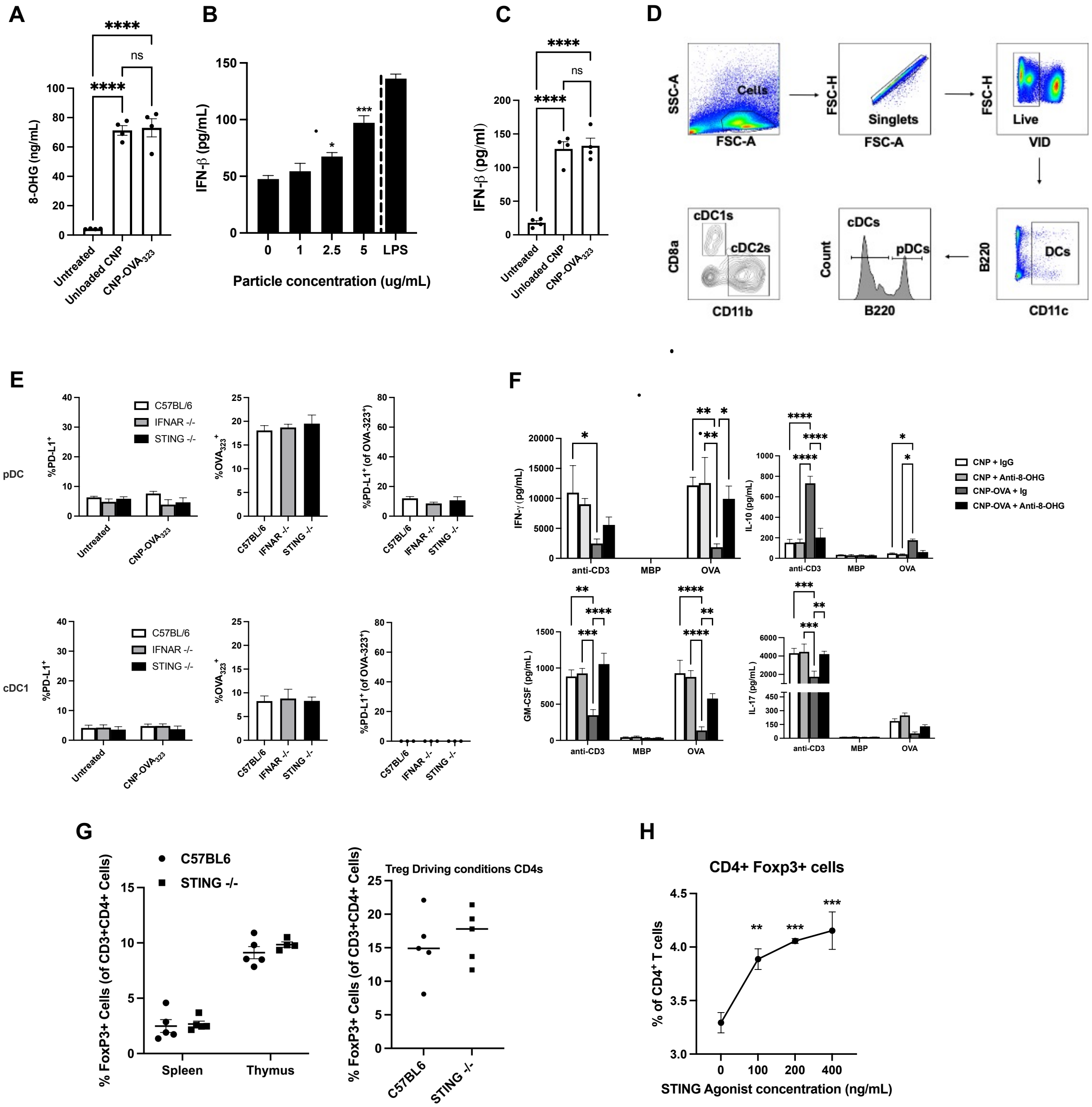

Supplemental Figure 5. Type I IFNs are induced via STING signaling after CNP treatment and induce a tolerogenic signature. BMDMs were cultured with either Unloaded CNP or CNP-$OVA_{323-339}$ for 30 minutes and the level of 8-OHG present within the culture supernatants was quantified via ELISA (**A**). BMDMs were treated with increasing concentrations of Unloaded CNPs or LPS for 24 hours and IFN-β was measured via ELISA (**B**). BMDMs were cultured with either Unloaded CNP or CNP-$OVA_{323-339}$ for 24 hours and IFN-β was measured via ELISA (**C**). Representative flow gating scheme for identifying pDCs, cDC1s, and cDC2s via flow cytometry for **Figure 6E** and **Supplemental Figure 5E** (**D**). The level of PD-L1 and $OVA_{323-339}$ present on the surface of pDCs and cDC1s was determined for the experiment presented in **Figure 6E** (**E**). For the DTH data presented in **Figure 7A**, *ex vivo* recall responses from total splenocytes was completed. Total splenocytes ($5x10^5$ cells/well) were cultured in the presence of anti-CD3 (1 μg/ml), OVA, or MBP (20 μg/ml) and culture supernatants were collected on Day 3 of culture. The level of secreted IFN-γ, IL-17, IL-10, and GM-CSF were measure via Luminex (**F**). The frequency of Treg cells present within the spleen and thymus of naïve C57BL6 and $STING^{-/-}$ mice (7–8-week-old female mice) was compared. Splenocytes from C57BL6 and $STING^{-/-}$ mice (7–8-week-old female mice) were cultured in the presence of IL-2 (100 U/ml), TGF-β (10 ng/ml), anti-CD3, anti-IFN-γ, anti-IL-4, and anti-IL-12 (1 μg/ml) for 72 hours, and the percentage of Treg cells analyzed via flow cytometry (**G**). Splenocytes from 5B6 mice were cultured in the presence of $PLP_{139-151}$ (20 μg/mL) and increasing concentrations of the STING agonist G3-YSD. The frequency of Tregs was quantified at 72 hours via flow cytometry (**H**). One representative experiment of two is presented Asterisks indicate a statistically significant difference as indicated by the bars *p <0.05, **p < 0.01, ***p < 0.001, and ****p < 0.0001, respectively.

Supplemental Table 1. Flow Cytometry Antibodies		
**Cell marker**	**Clone**	**Vendor**
**iNOS**	CXNFT	Invitrogen
**Perforin**	eBioOMAK-D	eBioscience
**MHCII**	NIMR-4	eBioscience
**IFNg**	XMG1.2	Invitrogen
**IL-15**	Polyclonal	AssayPro
**Il-10**	JES5-16E3	eBioscience
**Foxp3**	FJK-16S	Invitrogen
**CCL3**	DNT3CC	Invitrogen
**Arginase**	A1exF5	eBioscience
**CD244**	eBio244F4	eBioscience
**CD40**	3/23	BioLegend
**CD25**	PC61.5	eBioscience
**OX-40**	RM134L	BD
**Streptavidin**		eBioscience
**IL-1b**	NJTEN3	Invitrogen
**IL-10**	JES5-16E3	BioLegend
**IL-17A**	eBio17B7	Pharmingen
**F4/80**	BM8	eBioscience
**Granzyme B**	NG2B	eBioscience
**CD44**	IM7	BD
**VLA4**	R1-2	eBioscience
**Ly-6C**	AL-21	BD
**CD45**	30-F11	BD
**Lag3 (CD223)**	C9B7W	BD
**IFNg**	XMG1 .2	eBioscience
**CD206**	MR6F	eBioscience
**NKG2D (CD314)**	CX5	BD
**CD86**	GL-1	BioLegend
**PD-1 (CD279)**	J43	BD
**PD-L2**	TY25	BioLegend
**IL-12**	C17.8	eBioscience
**CTLA4 (CD152)**	UC10-4B9	eBioscience
**CD45**	RM4-5	BD
**Ki67**	16A8	BioLegend
**CD8**	53-6.7	BD
**CD80**	16-10A1	BD
**PD-L1 (CD274)**	MIH5	BD
**TNFa**	MP6-XT22	BD
**CD4**	RM4-5	BD
**CD11c**	HL3	BD
**CD3**	145-2C11	BD
**CD11b**	M1/70	BD
**NK1.1**	PK136	BD
**CD44**	IM7	BD
**CD25**	PC61	BD
**Ly-6G**	1A8	BD

Supplementary Table 1. List of flow cytometry antibodies. The table lists the antibodies used for the flow cytometry analysis, which includes both the clone and vendor for each respective antibody.
